# Transcriptomic analysis reveals the regulatory role of quorum sensing in the *Acinetobacter baumannii* ATCC 19606 via RNA-seq

**DOI:** 10.1186/s12866-022-02612-z

**Published:** 2022-08-16

**Authors:** Li Xiong, Fanli Yi, Qiuju Yu, Xiyue Huang, Keping Ao, Yuanfang Wang, Yi Xie

**Affiliations:** Department of Laboratory Medicine, West China Hospital, Sichuan University, Chengdu, China

**Keywords:** *Acinetobacter baumannii*, Quorum sensing, Regulatory mechanism, RNA-seq, Transcriptomic analysis, Virulence

## Abstract

**Background:**

*Acinetobacter baumannii* has emerged as the major opportunistic pathogen in healthcare-associated infections with high-level antibiotic resistance and high mortality. Quorum sensing (QS) system is a cell-to-cell bacterial communication mediated by the synthesis, secretion, and binding of auto-inducer signals. It is a global regulatory system to coordinate the behavior of individual bacteria in a population. The present study focused on the QS system, aiming to investigate the regulatory role of QS in bacterial virulence and antibiotic resistance.

**Method:**

The auto-inducer synthase gene *abaI* was deleted using the *A. baumannii* ATCC 19606 strain to interrupt the QS process. The RNA-seq was performed to identify the differentially expressed genes (DEGs) and pathways in the mutant (△abaI) strain compared with the wild-type (WT) strain.

**Results:**

A total of 380 DEGs [the adjusted *P* value < 0.05 and the absolute value of log_2_(fold change) > log_2_1.5] were identified, including 256 upregulated genes and 124 downregulated genes in the △abaI strain. The enrichment analysis indicated that the DEGs involved in arginine biosynthesis, purine metabolism, biofilm formation, and type VI secretion system (T6SS) were downregulated, while the DEGs involved in pathways related to fatty acid metabolism and amino acid metabolism were upregulated. Consistent with the expression change of the DEGs, a decrease in biofilm formation was observed in the △abaI strain compared with the WT strain. On the contrary, no obvious changes were found in antimicrobial resistance following the deletion of *abaI*.

**Conclusions:**

The present study demonstrated the transcriptomic profile of *A. baumannii* after the deletion of *abaI*, revealing an important regulatory role of the QS system in bacterial virulence. The deletion of *abaI* suppressed the biofilm formation in *A. baumannii* ATCC 19606, leading to decreased pathogenicity. Further studies on the role of *abaR*, encoding the receptor of auto-inducer in the QS circuit, are required for a better understanding of the regulation of bacterial virulence and pathogenicity in the QS network.

**Supplementary Information:**

The online version contains supplementary material available at 10.1186/s12866-022-02612-z.

## Background

*Acinetobacter baumannii* is a leading primary opportunistic pathogen responsible for healthcare-associated infections; it causes a variety of clinical infections such as pneumonia, and infections of the bloodstream, central nervous system, urinary tract, skin, and soft tissue [1, 2]. *A. baumannii* infections are difficult to treat owing to high-level antibiotic resistance and high mortality [3, 4], necessitating the unraveling of the mechanism of bacterial virulence and developing novel antimicrobial agents. *A. baumannii* harbors various virulence factors for functions such as resistance to desiccation, biofilm formation, motility, secretion systems, surface glycoconjugates, and micronutrient acquisition systems [5–7]. Several studies linked these virulence factors to the quorum sensing (QS) system, which is a cell-to-cell bacterial communication mediated by the auto-inducer to maintain cell density, indicating that QS might be a regulatory pathway implicated in bacterial virulence [8–11].

The QS circuit in *A. baumannii* comprises the *abaI* encoding auto-inducer synthase and *abaR* encoding the cognate receptor of the auto-inducer [12]. *A. baumannii* can produce and secrete various auto-inducers, of which the major signal is N-hydroxydodecanoyl-L-homoserine lactone (OHC12-HSL), a long-chain N-acyl-homoserine lactone [13]. When the concentration of the auto-inducers reaches a certain threshold during bacterial growth, the auto-inducers bind to AbaR, subsequently altering the expression of the downstream genes, which further regulates the biological processes. Previous studies reported the involvement of QS in regulating the motility, biofilm formation, and antibiotic resistance in bacteria [9–11, 14]. The deletion of *abaI* attenuated the bacterial virulence in *Galleria mellonella* [11].

In the present study, *abaI* was knocked out using the type strain *A. baumannii* ATCC 19606, which was first isolated in 1948 from the urine of a patient with urinary infection and is widely used in studying the virulence, pathogenesis, and antimicrobial resistance as a reference and model organism [15]. The whole genome of ATCC 19606 comprises a 3,981,941-bp chromosome and two plasmids with an average GC content of 39.15% [16]. The RNA sequencing was performed to identify the differentially expressed genes (DEGs) and pathways in the mutant (△abaI) strain compared with the wild-type (WT) strain, providing a better understanding of the regulatory mechanism of bacterial virulence and drug resistance at the genetic level.

## Results

### Differentially expressed genes

**Fig. 1:**
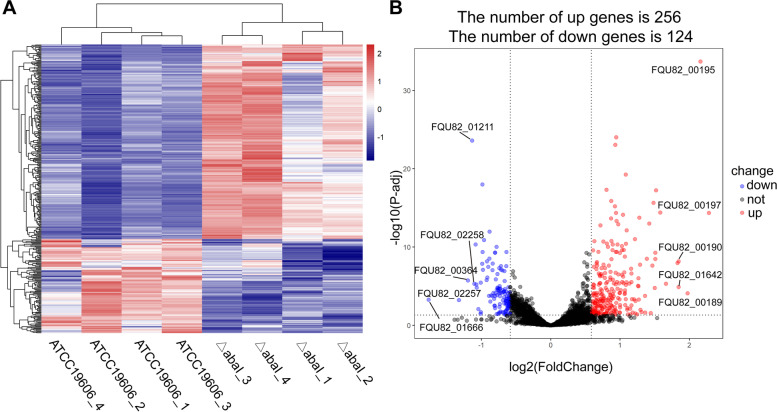
Differentially expressed genes (DEGs) identified in the mutant strain △abaI compared with the wild-type strain. **A** Expression heatmap of 380 DEGs by hierarchical clustering, with red representing the relatively higher level of expression and blue representing the relatively lower level of expression. **B** Volcano plot of DEGs with 256 upregulated genes (red dot) and 124 downregulated genes (blue dot). The black dots refer to the genes with no significant expression change. The top five up- and downregulated DEGs are marked in the plot

A total of 31.5 and 30.5 million reads on average were obtained from the △abaI and the WT strains using sequencing. The cleaned and aligned ratios were 94%–99% and 97%–99%, respectively (Table 1). The principal component analysis (PCA) (Fig. S1) showed that the △abaI and the WT strains were clustered into two groups based on the gene expression matrix, indicating a good replication of raw data among the samples. After differential expression analysis, 380 DEGs [*P*-adj < 0.05 and the absolute value of log_2_(fold change) > log_2_1.5] were identified, including 256 upregulated genes and 124 downregulated genes in the △abaI strain compared with the WT strain (Fig. 1). These DEGs included 65 upregulated genes with fold change (FC) > 2 and 12 downregulated genes with FC < 0.5.

**Table 1:** Cleaned and aligned status of the raw reads

Sample	Total reads	Clean reads	Mapped reads	Pair mapped reads	Single mapped reads	Mapped ratio
△abaI_1	31,797,602	30,740,582	29,903,793	29,803,250	100,543	97.28%
△abaI_2	31,527,344	30,577,998	29,770,300	29,669,278	101,022	97.36%
△abaI_3	31,343,136	30,159,134	30,600,276	30,071,724	87,410	98.56%
△abaI_4	31,405,322	30,469,784	29,686,661	29,597,664	88,997	97.43%
ATCC19606_1	31,114,984	30,482,962	30,140,345	30,063,360	76,985	98.88%
ATCC19606_2	30,122,764	29,709,768	29,321,240	29,231,694	89,546	98.69%
ATCC19606_3	30,476,618	29,154,504	28,372,156	28,283,012	89,144	97.32%
ATCC19606_4	30,307,370	28,710,874	28,373,203	28,296,826	76,377	98.82%

### Clusters of orthologous groups functional classification

**Fig. 2:**
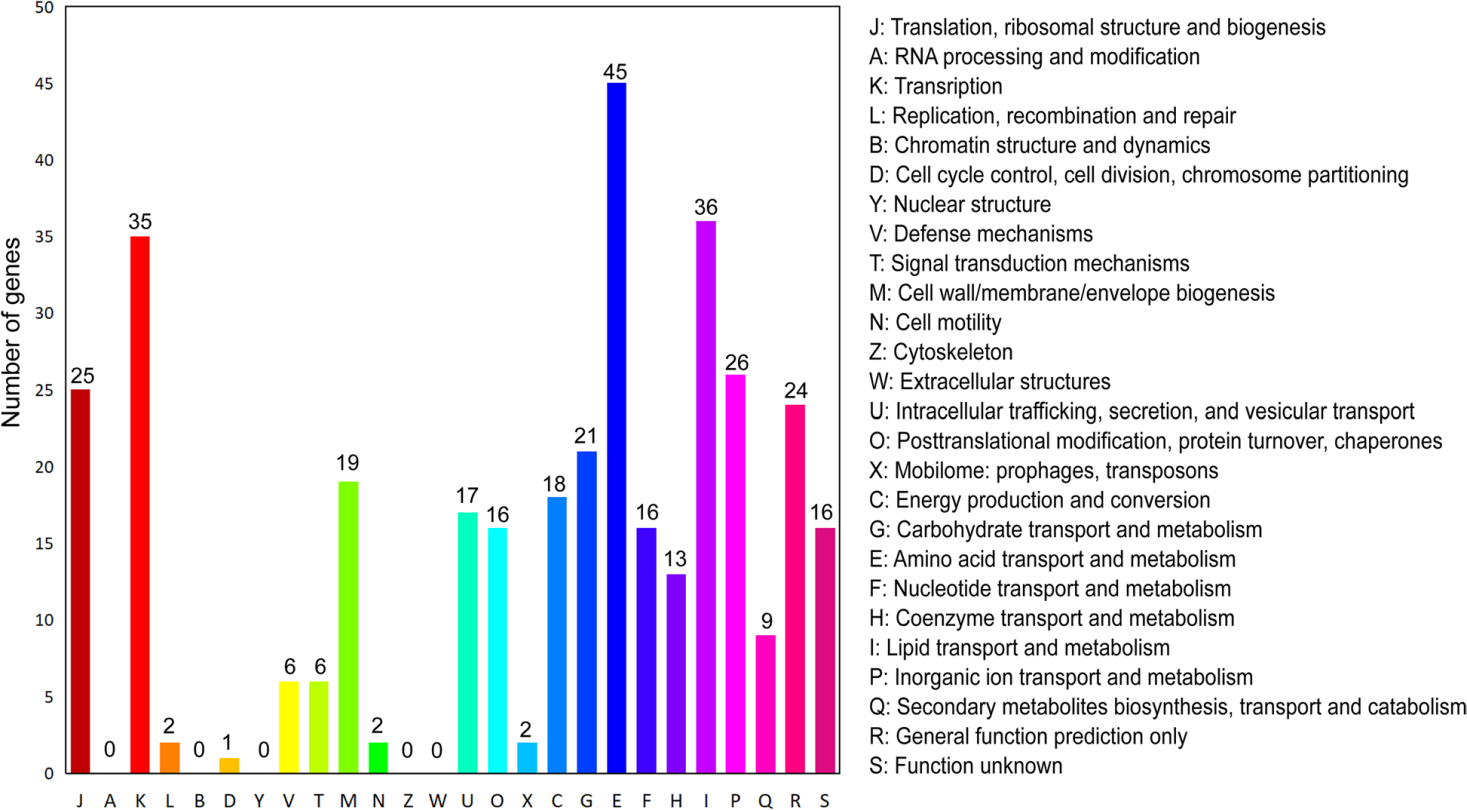
Clusters of Orthologous Groups (COG) enrichment of DEGs, which were divided into functional categories by alignment to the latest COG database

The DEGs were annotated and clustered into the Clusters of Orthologous Groups (COG) functional categories through ortholog alignment of the amino acid sequence to the COG database (Fig. 2). Among the DEGs, 12.7% were classified into [E] amino acid transport and metabolism, 10.1% into [I] lipid transport and metabolism, 9.9% into [K] transcription, 7.3% into [P] inorganic ion transport and metabolism, 7.0% into [J] translation, ribosomal structure, and biogenesis, 6.8% into [R] general function prediction only, and 5.9% into [G] carbohydrate transport and metabolism with gene number more than 20.

### Gene ontology analysis

**Fig. 3:**
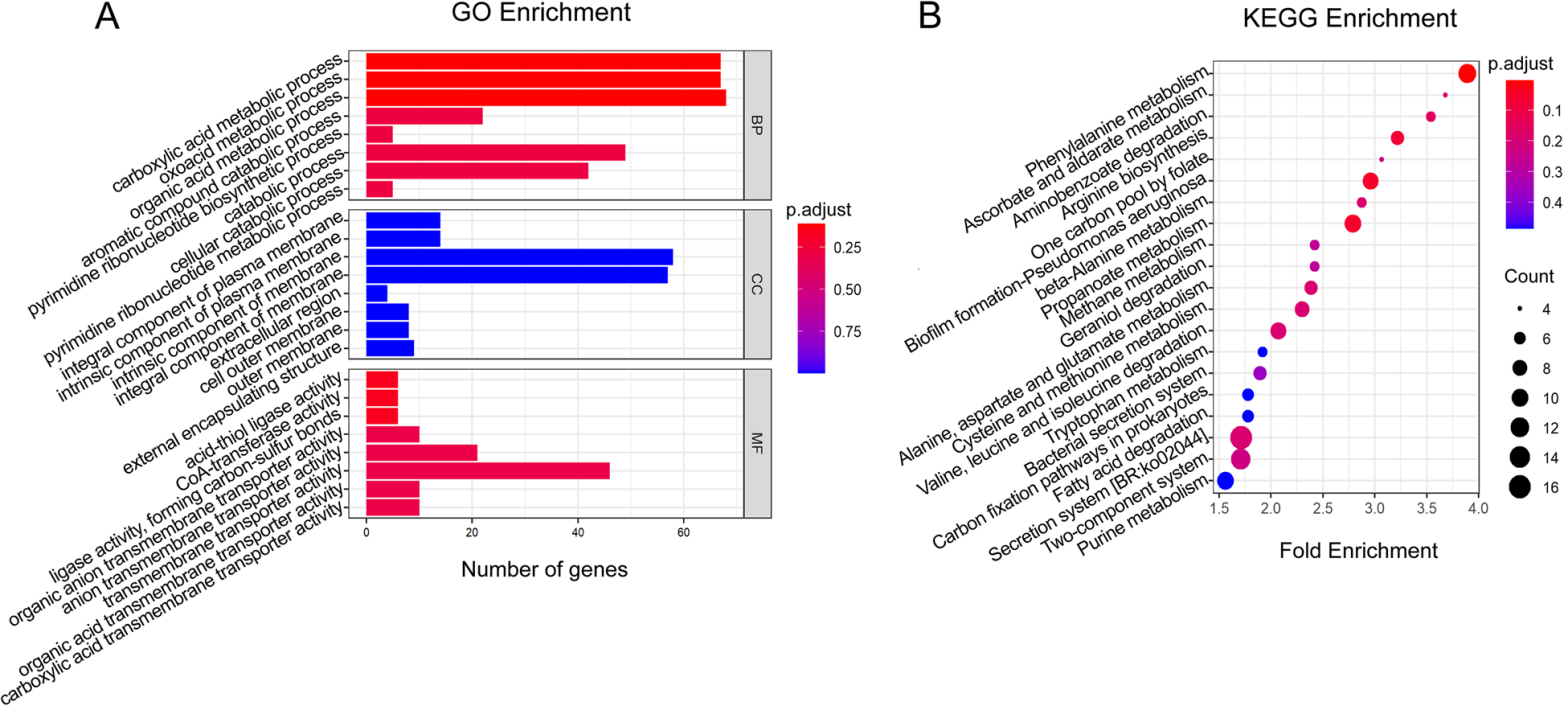
GO and KEGG enrichment of DEGs. **A** Top eight GO enrichment terms in each ontology group included biological process, cellular component, and molecular function. The *P*-adjusted value of each term is shown as a color gradient. **B** Top 20 KEGG pathways of DEGs with fold enrichment (GeneRatio/BgRatio) on the *x*-axis. The number of genes enriched in each KEGG pathway is shown as the size of the dot, and the *P-*adjusted value is shown as a color gradient of the dot

After Gene Ontology analysis, the DEGs were annotated and enriched into three physiological function categories: biological process, cellular component, and molecular function (Fig. 3A). In biological process, the mainly enriched functional categories comprised organic acid metabolic process, aromatic compound catabolic process, cellular catabolic process, and pyrimidine ribonucleotide biosynthetic and metabolic process. In cellular component, the mainly enriched functional categories were intrinsic component of membrane, extracellular region, and external encapsulating structure. In molecular function, the mainly enriched functional categories were CoA-transferase activity, ligase activity forming carbon-sulfur bonds, and transmembrane transporter activity for anions and organic acids.

### Kyoto encyclopedia of genes and genomes pathway enrichment

Kyoto Encyclopedia of Genes and Genomes (KEGG) is a general and abundant database meant for the biological interpretation of genes and genomes. The KEGG enrichment was analyzed based on the alignment of query amino acid sequences to the KO database to investigate the effect of QS on the molecular interaction network and pathway in *A. baumannii*. The DEGs were mainly involved in the pathways related to amino acid metabolism, biofilm formation, fatty acid metabolism, secretion system, two-component system, and purine metabolism (Fig. 3B).

### Amino acid metabolism

In amino acid metabolism pathways, the partial DEGs were involved in metabolizing phenylalanine, arginine, cysteine, methionine, alanine, aspartate, glutamate, valine, leucine, and isoleucine. The phenylalanine metabolism was associated with bacterial growth and virulence [17–19]. In the △abaI strain, the upregulated DEGs involved in this pathway included the FQU82_00157, FQU82_00183, FQU82_02324, FQU82_01642(*paaF*), FQU82_02187, FQU82_01589(*paaK*), FQU82_01581(*paaB*), FQU82_01580(*paaA*), and FQU82_01583(*paaD*), while FQU82_02901 was found to be downregulated.

Among the DEGs involved in the arginine biosynthesis, FQU82_00067 and FQU82_01752 were upregulated, whereas FQU82_01177, FQU82_02901, FQU82_01050 (*ureC*), FQU82_01049 (*ureB*), and FQU82_01048 (*ureA*) were downregulated. Arginine was one of the common amino acids required for protein synthesis in bacteria, constituting about 5% of the total proteins of *Escherichia coli* [20, 21].

Valine, leucine, and isoleucine were branched-chain amino acids related to the growth and virulence of the bacteria [22, 23]. Nine DEGs were enriched in the degradation of valine, leucine, and isoleucine, and all of them were upregulated.

### Biofilm formation

The DEGs enriched in the biofilm formation pathway comprised nine downregulated genes: FQU82_01545 (*tagF*), FQU82_01550 (*tssK*), FQU82_015379 (*tssB*), FQU82_01549 (*tssA*), FQU82_01538 (*tssC*), FQU82_01544 (*tssM*), FQU82_01548 (*tssH*), FQU82_01539 (*hcp*), and FQU82_01542 (*tssG*). These genes were also associated with the type VI secretion system.

### Secretion system

In this study, 16 downregulated DEGs were found to be enriched in the secretion system pathway. Among these genes, 14 were involved in the type VI secretion system: FQU82_01545 (*tagF*), FQU82_01550 (*tssK*), FQU82_01537 (*tssB*), FQU82_01549 (*tssA*), FQU82_01538 (*tssC*), FQU82_01544 (*tssM*), FQU82_01548 (*tssH*), FQU82_03692 (*vgrG2a*), FQU82_01551 (*tssL*), FQU82_01540 (*tssE*), FQU82_01539 (*hcp*), FQU82_00952 (*vgrG2b*), FQU82_01541 (*tssF*), and FQU82_01542 (*tssG*). FQU82_00545 (*tatB*) was involved in the twin-arginine translocation system, and FQU82_01811 was involved in the type 1 pilus assembly.

### The two-component system

The upregulated DEGs were found to be enriched in the two-component system, including FQU82_00307 (*pilS*), FQU82_01779, FQU82_02017, FQU82_01778, FQU82_01780, FQU82_01752, FQU82_02461 (*kdpA*), and FQU82_02460 (*kdpB*). The downregulated DEGs in this pathway included FQU82_02258 (*cydA*), FQU82_02259 (*cydB*), FQU82_02260 (*cydX*), FQU82_02840, and FQU82_02515. Among these genes, FQU82_00307(*pilS*) was a sensor histidine kinase of the PilS/PilR system, which was associated with the type-4 fimbriae synthesis contributing to bacterial motility [24, 25].

### Propanoate and purine metabolism

Propanoate and purine metabolism are both critical metabolic pathways related to growth and virulence in bacteria [26, 27]. In this study, 10 upregulated DEGs were found to be enriched in the propanoate metabolism: FQU82_00191, FQU82_00562, FQU82_03635, FQU82_01642 (*paaF*), FQU82_00192, FQU82_00189 (*mmsA*), FQU82_00193, FQU82_00159 (*prpB*), FQU82_00160 (*prpC*), and FQU82_00161 (*acnD*). In purine metabolism, 8 DEGs were downregulated, including FQU82_00576 (*ndk*), FQU82_02944 (*purL*), FQU82_02508 (*purH*), FQU82_02833 (*purB*), FQU82_00036, FQU82_01050 (*ureC*), FQU82_01048 (*ureA*), and FQU82_01049, and 2 were upregulated, including FQU82_02113 and FQU82_00719. A key gene in purine metabolism FQU82_00576 (*ndk*), encoding an evolutionarily conserved NTP-generating kinase, was vital for nucleic acid biosynthesis [27].

### Gene set enrichment analysis

**Fig. 4:**
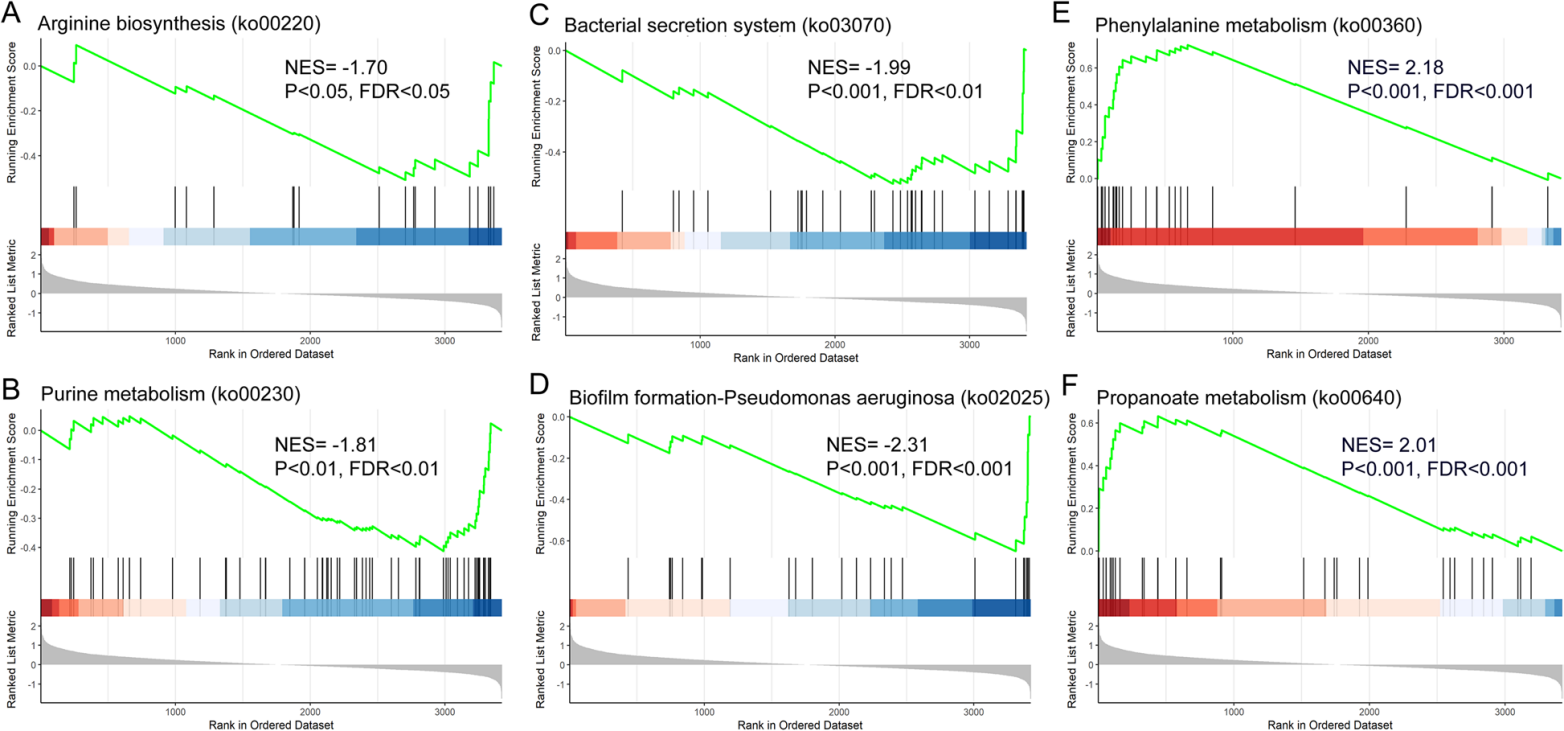
Gene set enrichment analysis of the KEGG pathways based on the ordered gene expression profile in general. **A**–**F** Enrichment plot of the arginine biosynthesis, purine metabolism, bacterial secretion system, biofilm formation, phenylalanine metabolism, and propanoate metabolism

Gene set enrichment analysis (GSEA) is a bioinformatics tool for interpreting genome-wide expression profiles at the level of gene sets [28]. The GSEA analysis was conducted to determine the general expression trend of enriched pathways in the KEGG analysis. The results showed that the genes enriched in the phenylalanine metabolism and propanoate metabolism were concentrated at the top of the ordered gene expression set. The normalized enrichment scores (NESs) were 2.18 and 2.01, respectively, with FDR < 0.001 (Fig. 4E and F), indicating that the whole gene sets in these pathways were upregulated in general. However, the genes enriched in the arginine biosynthesis, purine metabolism, bacterial secretion system, and biofilm formation were distributed at the bottom of the ordered gene expression profile (NES = –1.70, –1.81, –1.99, and –2.31, respectively, FDR < 0.05) (Fig. 4A–D), indicating that the whole gene sets in these pathways were downregulated in general.

### Validation of RNA sequencing

**Fig. 5:**
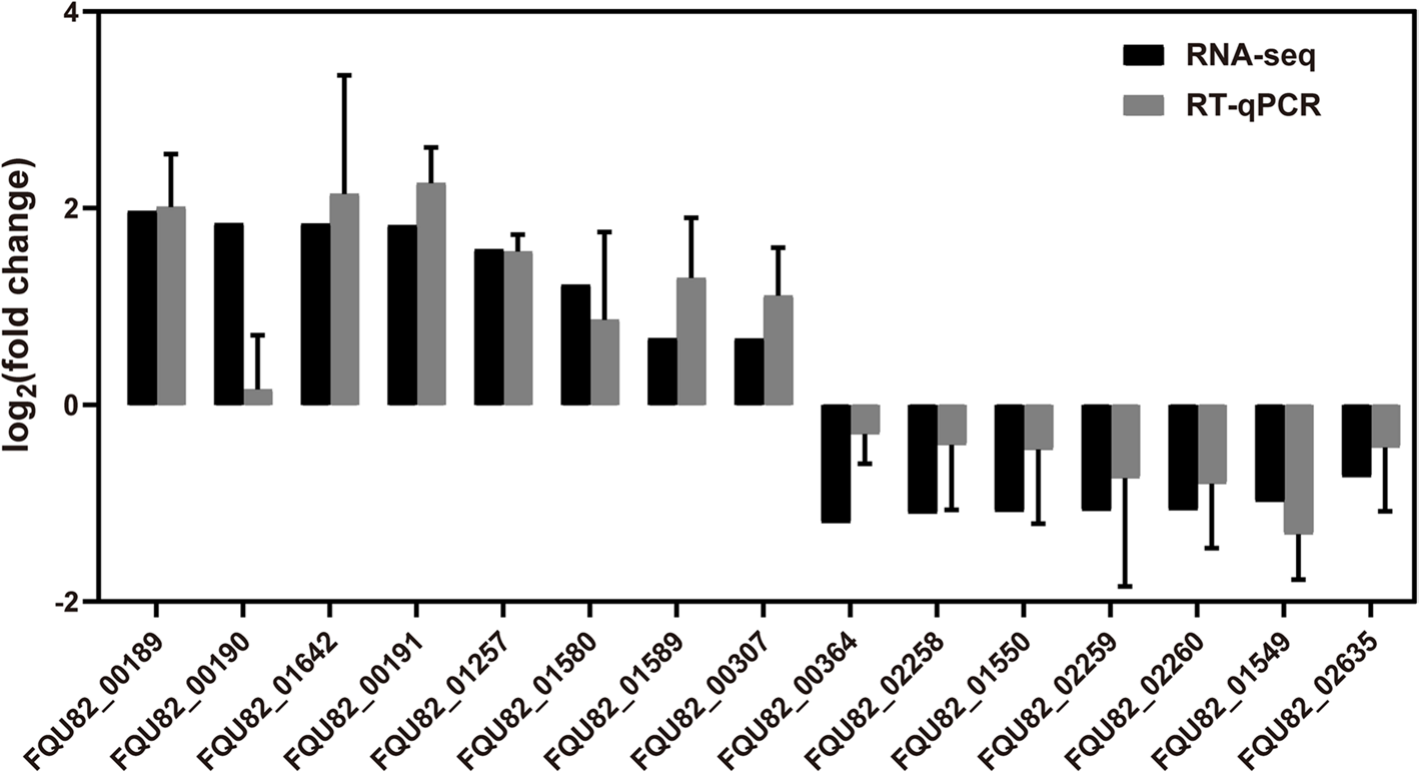
Validation of RNA sequencing using the real-time qPCR. Four individual biological replicates were performed in triplicates. The data represented the means ± standard deviation of the results

A total of 15 genes were subjected to reverse transcription quantitative polymerase chain reaction (RT-qPCR), including 10 genes selected from the top up- and downregulated DEGs and another 5 DEGs, to confirm the reliability of RNA sequencing and transcriptomic analysis (Fig. 5). The expression trend of these genes was consistent in RT-qPCR and RNA sequencing, indicating the reliability of the expression data by RNA sequencing. The disparity between RNA-seq and RT-qPCR was possibly attributed to the small sample number, RNA degradation, and low expression level of genes.

### Antibiotic resistance profile and biofilm formation

The minimum inhibitory concentrations (MICs) of 16 commonly used antibiotics were detected to determine whether the deletion of *abaI* changed the antibiotic resistance. The results showed that the mutant strain △abaI was more resistant to cefepime compared with the WT strain with a slightly increased MIC. The interpretive category of MIC results turned to intermediate (I) from susceptible (S) after *abaI* deletion. However, the MIC of amikacin decreased following the deletion of *abaI*, with no change in the interpretation of the susceptibility to this drug (Table 2).

**Table 2:** Antibiotic resistance profile of the strain used

Antibiotic	WT		△abaI	
	MIC (μg/mL)	Interpretation	MIC (μg/mL)	Interpretation
Ampicillin	≥ 32	R	≥ 32	R
Piperacillin	16	S	16	S
Piperacillin/Tazobactam	8	S	8	S
Cefazolin	≥ 64	R	≥ 64	R
Ceftazidime	8	S	8	S
Ceftriaxone	16	I	16	I
Cefepime	8	S	16	I
Aztreonam	32	R	32	R
Imipenem	≤ 0.25	S	≤ 0.25	S
Meropenem	1	S	1	S
Amikacin	4	S	≤ 2	S
Gentamicin	4	S	4	S
Ciprofloxacin	≥ 4	R	≥ 4	R
Levofloxacin	≥ 8	R	≥ 8	R
Tetracycline	≥ 16	R	≥ 16	R
Tigecycline	1	S	1	S

**Fig. 6:**
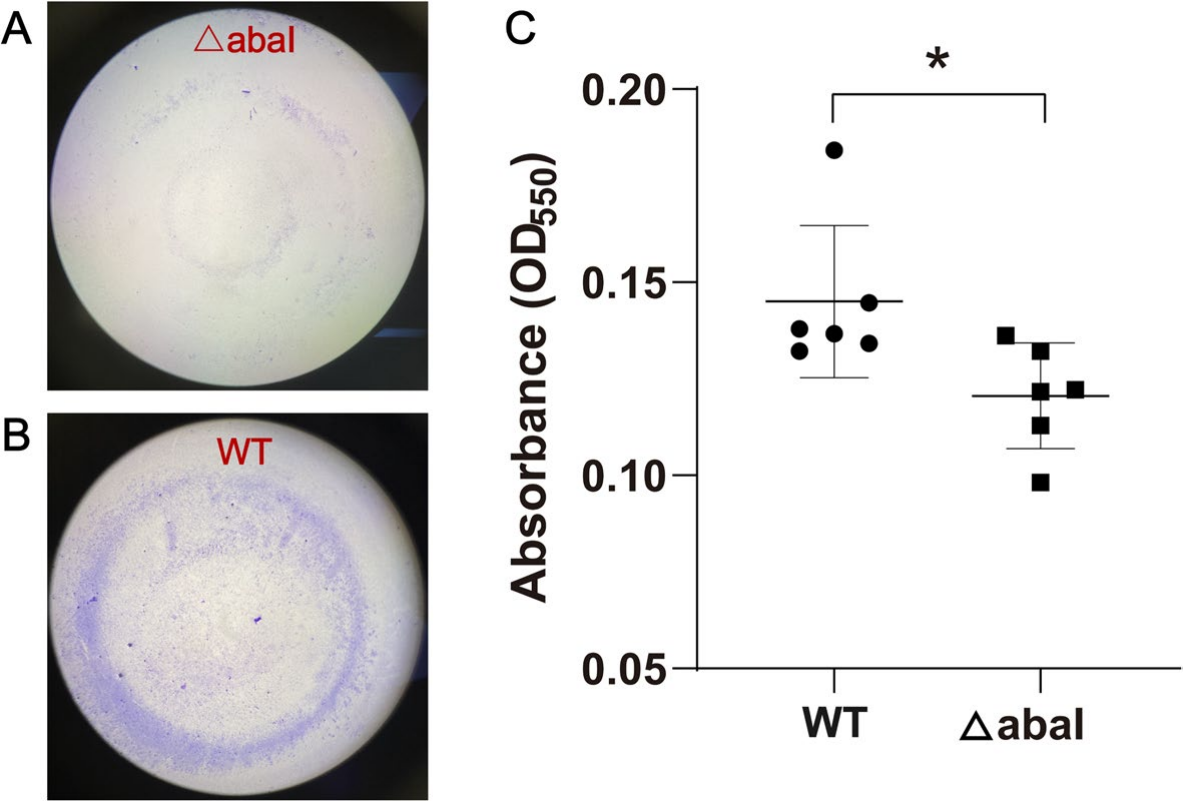
Biofilm formation assay of wild-type (WT) and mutant (△abaI) strains. **A** and **B** Image of the biofilm of WT and △abaI strains stained using CV staining in a 96-well plate after 24-h incubation at 37℃ (original magnification × 40). **C** Absorbance of CV solution at 550 nm between WT and △abaI strains. Six individual biological replicates were performed in quadruplicates, and the data were represented as the means ± standard deviation of the results. ^*^P < 0.05

With respect to biofilm formation, crystal violet (CV) staining showed that the biofilm formation ability decreased following the deletion of *abaI*, which was consistent with the expression change of the DEGs. As shown in Fig. 6, the stained biofilm of the WT strain presented an obvious purple circle, while the stained area of the △abaI strain was fragmented after an incubation time of 24 h. The absorbance of CV solution also decreased significantly in the △abaI strain compared with the WT strain.

## Discussion

Recently, QS was discovered as a new potential antimicrobial target in *A. bauammii* [12, 29, 30]. Multiple studies demonstrated that quorum quenching reduced the QS-mediated virulence of bacteria by inhibiting QS [10, 31, 32]. A mutant strain with *abaI* (auto-inducer synthases) knockout in the *A. baumannii* ATCC 19606 strain was generated, and RNA-seq was performed to investigate the regulatory role of QS in bacterial pathogenicity. As a result, 256 upregulated and 124 downregulated DEGs were identified via the transcriptomic analysis. In previous studies, the RNA-seq was performed in the *abaI* deletion mutant of several *A. baumannii* strains, including the *A. baumannii* ATCC 17978, AB5075, and clinical strains [11, 33, 34]. The transcriptomic analysis revealed that the number of DEGs varied due to different cutoffs of log_2_ (FC) and culture conditions. A previous study showed that the concentration of AHLs peaked around 17–24 h of culture, and the low-salt culture condition increased the concentration of OHC12-HSL and expression of *abaI* [13], influencing the expression of QS-related downstream genes. These genes were primarily the regulators of various biological processes associated with bacterial growth and virulence, including biofilm formation, secretion system, and biosynthesis and metabolism of fundamental substances.

The transcriptomic data revealed that some genes associated with bacterial virulence were downregulated in the △abaI strain, including biofilm formation, type VI secretion system (T6SS), and two-component system. The DEGs enriched in the biofilm formation were simultaneously involved in T6SS, an important virulence factor. Several studies showed that T6SS contributed to biofilm formation, surface motility, bacterial competition, virulence, and antibiotic resistance of *A*. *baumannii* [8, 35–38]. The genes enriched in this system were responsible for encoding the basic structures of the functional T6SS system, including *vgrG*, *hcp*, and *tss* cluster (Fig. S3B). These genes were downregulated in the △abaI strain, indicating that QS played a positive regulatory role in T6SS. Moreover, FQU82_00545 (*tatB*) and FQU82_01811 were involved in the twin-arginine translocation system and type 1 pilus assembly, which were essential for the export of the folded proteins, bacterial colonization, and biofilm formation [39, 40]. In our previous study, the *csuA/BABCDE* chaperone-usher system enhanced the pili assembly in the *A. baumannii* ATCC 19606 strain when co-cultured with a QS signaling molecule C6-HSL [41]. The transcriptomic analysis showed increased expression of *csuA*, *csuB*, *csuC*, *csuD*, and *csuE* with FC more than 2, indicating that QS played an important role in the regulation of bacterial biofilm formation. Consistent with these observed DEGs, the biofilm formation ability was inhibited in the △abaI strain compared with the WT strain, which was similar to the findings of other studies [11, 34].

Regarding substance metabolism, the genes involved in arginine biosynthesis and purine metabolism were downregulated following the deletion of *abaI*. Both arginine and purine were the basic source of protein and nucleic acid biosynthesis. The attenuated utilization of the basic substances might limit bacterial growth, leading to the suppressed growth curve of the *abaI*-deleted strain observed in previous studies [11, 33]. On the contrary, the gene set of phenylalanine metabolism and propanoate metabolism displayed an upregulated trend in the △abaI strain, similar to previous findings [11]. As shown in the KEGG pathway map of phenylalanine metabolism (Fig. S3A), the degradation of phenylacetate was enhanced by an upregulated paa operon (*paaA*, *paaB*, *paaD*, *paaJ*, *paaK*, and *paaF*), increasing the levels of acetyl-CoA and succinyl-CoA. In addition, the expression profile of the enriched DEGs of propanoate metabolism was also enhanced in the acetyl-CoA and succinyl-CoA synthesis (Fig. S4). The accumulation of acetyl-CoA and succinyl-CoA in both phenylalanine metabolism and propanoate metabolism was related to bacterial physiology and virulence via lysine acetylation, which was a post-translational modification [42, 43].

Furthermore, other DEGs were found to be vital for bacterial virulence and drug resistance. The downregulated gene FQU82_00521(*tonB*_*3*_) was essential for active iron uptake in *A. buamannii,* contributing to bacterial pathogenicity [44]. The present study showed several DEGs related to drug resistance, such as the upregulated gene FQU82_03438 (*tetA*) encoding a tetracycline transporter protein. The overproduction of TetA conferred resistance to tigecycline [45]. A previous study showed that the △abaI strain was more resistant to tetracycline in *A. baumannii* ATCC 17978 [11]. However, no change was found in the resistance of the △abaI strain to tetracycline in *A. baumannii* ATCC 19606. Besides, the multidrug resistance gene FQU82_03743 (*norM*) was upregulated in the △abaI strain, indicating a slight increase in the MIC of cefepime observed in the △abaI strain.

## Conclusions

Collectively, the present study indicated that the deletion of *abaI* attenuated bacterial virulence, including biofilm formation and T6SS. It was presumed that targeting the inhibition of QS might serve as a possible strategy for designing new antimicrobial agents. However, further studies regarding *abaR*, the receptor of auto-inducer in the QS circuit, are required to illustrate the detailed regulation mechanism of QS.

## Materials and methods

### Bacterial strains and culture conditions

The bacterial strains and plasmids used in this study are listed in Table 3. The strains were cultured in the Luria–Bertani (LB) medium at 37℃.

**Table 3:** Bacterial strains and plasmids used in this study

Strain or Plasmid	Description	Reference or Source
*A. baumannii*		
ATCC 19606	Wild type (WT)	ATCC
△abaI	WT with a deletion in *abaI* gene	This study
*Escherichia coli*		
DH5α	Competent cells for plasmid cloning	Sangon Biotech Co., Ltd
β2155	Donor strain for plasmid transformation	Sangon Biotech Co., Ltd
Plasmid		
pKD4	Vector plasmid of Kanamycin-resistant gene	Addgene
pCVD442	Suicide plasmid for target gene deletion	Addgene

### Construction of the *abaI* deletion strain

The △abaI strain was constructed by the suicide plasmid-mediated gene knockout method at Sangon Biotech Co., Ltd. (Shanghai, China). Briefly, the upstream and downstream homologous arms of the target gene were connected with the kanamycin-resistant gene amplified from the plasmid pKD4 by fusion PCR. The fusion fragment was ligated into the suicide vector pCVD442 to obtain the targeting plasmid pCVD442-△abaI::Kn and subsequently introduced into the *E. coli* DH5α through electroporation. The *E. coli* DH5α was cultured in the LB medium containing 50 μg/mL ampicillin and 20 μg/mL kanamycin at 37℃, and the ampicillin- and kanamycin-resistant colonies carrying pCVD442-△abaI::Kn were selected to purify the plasmid via column centrifugation. Then, the purified plasmid was electrotransferred into *E. coli* β1255 and cultured in the LB medium containing 100 μg/mL ampicillin and 0.5 mM meso-2,5-diaminopimelic acid. The ampicillin-resistant colonies were selected to be conjugated with *A. baumannii* ATCC 19606 as the donor strain. The conjugated strains were cultured in the LB medium containing 33 μg/mL kanamycin, and the kanamycin-resistant *A. baumannii* colonies were screened and cultured in the LB medium containing 10% sucrose and 33 μg/mL kanamycin. Finally, the kanamycin-resistant *A. baumannii* strain was selected to perform PCR amplification and sequencing for verifying the target gene. The *A. baumannii* colony, whose *abaI* gene was replaced with the kanamycin-resistant gene, was used as the *abaI* deletion strain △abaI*.*

### RNA extraction and sequencing

The *A. baumannii* ATCC 19606 and △abaI strains were cultured on the blood agar plate overnight at 37℃. The colonies were suspended in sterile saline, with the turbidity set to 0.5 McFarland standard. Then, 100 μL of bacterial suspension was inoculated into 5 mL of LB broth and incubated at 37℃ for 12 h. The overnight cultures centrifuged at 10,000 rpm for 5 min and used to extract RNA. The total RNA was extracted from the harvested bacteria using an RNeasy mini kit (Qiagen, Germany) following the manufacturers’ protocols. The RNA quality and concentration were evaluated by measuring the absorbance and calculating the A260/A280 and A260/A230 ratios using a NanoDrop One Microvolume UV spectrophotometer (Thermo Scientific, MA, USA). The RNA integrity was evaluated using an Agilent 2100 Bioanalyzer (Agilent Technologies, CA, USA) and verified after gel electrophoresis. The samples with the RNA integrity number (RIN) ≥ 7 and 28S/18S ratio ≥ 0.7 were used for the subsequent analysis. The library was constructed using a SureSelect Strand-Specific RNA Component kit (Agilent, CA, USA) and evaluated with Qubit 3 Fluorometer (Thermo Scientific, MA, USA). The libraries were sequenced using Illumina NovaSeq 6000 (Illumina, CA, USA). Four biological replicates were performed in this study. All the RNA-seq data obtained in this study were submitted to the National Center for Biotechnology Information (NCBI) Sequence Read Archive with the accession number PRJNA770023.

### Transcriptomic analysis

The fastp software [46] was used to clean and evaluate the qualities of the raw reads from sequencing. The reads containing the adapter, poly-N, and more than 40% low-quality bases (Q < 20) were cleaned. The rRNA was removed using the Sortmerna software [47]. The cleaned reads were mapped onto the genome of the *A. baumannii* ATCC 19606 strain (GenBank accession no**. **CP045110.1) as the reference genome using bowtie2. Samtools and featurecounts [48] were used to count the aligned reads to genomic features for downstream analyses. The differential gene expression was analyzed using the DESeq2 package (Bioconductor) [49] based on the negative binomial distribution employing the R software. The genes with the adjusted *P* value (*P*-adj) < 0.05 and the absolute value of log_2_(FC) > log_2_1.5 were deemed as the DEGs. The principal component analysis (PCA), heatmap, and volcano maps were performed using the pheatmap and ggplot2 packages [50, 51]. The COG enrichment analysis was performed by classifying the DEGs into functional categories based on the similarity of the amino acid sequence. The GO enrichment, KEGG enrichment, and GSEA were conducted using the clusterProfiler package [52] to identify the biological functions and pathways mainly affected by the DEGs.

### Validation of RNA sequencing

The top 10 DEGs among the up- and downregulated genes and 5 DEGs of interest were analyzed using the two-step RT-qPCR with the same RNA-seq samples to validate the reliability of the RNA-seq results. The reverse transcription was conducted using the HiScript II Q RT SuperMix for the qPCR kit (Vazyme, China), and the real-time qPCR was conducted using an NuHi Robustic SYBR Green Mix kit (Nuhigh, China). The RT-qPCR was performed on an ABI Prism 7900 PCR system (Applied Biosystems, MA, USA) using the reaction as follows: initial denaturation at 95℃ for 10 min, followed by 40 cycles of reaction at 95℃ for 15 s and 60℃ for 60 s. The melting curve was plotted after the end of the cycle to verify the specificity of the amplification product. Each PCR reaction was repeated thrice. The gene expression was calculated and compared using the 2^−△△Ct^ method, with 16 s rRNA as an endogenous reference.

### Antimicrobial susceptibility testing

The MICs of 16 antibiotics, including ampicillin, piperacillin, piperacillin/tazobactam, cefazolin, ceftazidime, ceftriaxone, cefepime, aztreonam, imipenem, meropenem, amikacin, gentamicin, ciprofloxacin, levofloxacin, tetracycline, and tigecycline, were determined on an automatic microbial analyzer (Vitek 2 Compact, France) by the broth microdilution method. The results of MICs were interpreted according to the criteria mentioned in the Clinical and Laboratory Standards Institute (CLSI) M100 guidelines. All experiments were performed three times.

### Biofilm formation assay

The ability to form a biofilm of the strains was tested using CV staining following the previously described protocol with minor modifications [53]. Briefly, the overnight culture of the strain was suspended in sterile saline to turbidity comparable to a 0.5 McFarland standard. Then, 20 μL of the suspension was added to a 96-well microtiter plate containing 180 μL of the tryptic soy broth (TSB) with 1% glucose and incubated at 37℃ for 24 h. Following incubation, the contents of the wells were removed, and each well was washed three times with 300 μL of phosphate-buffered saline (PBS). Then, 200 μL of 4% paraformaldehyde (PFA) was added to increase biofilm stability for 20 min. Subsequently, each well was rinsed with 300 μL of PBS. PBS was removed as much as possible, and the contents were stained with 175 μL of 0.1% CV for 15 min. The wells were then rinsed three times with 300 μL of PBS and dried at room temperature overnight. Further, 175 μL of 95% ethanol was added to each well to solubilize the CV, and the optical density (OD) of all wells was measured in a microplate spectrophotometer at 550 nm. Six individual biological replicates were performed in quadruplicates. The differences in the OD value between WT and △abaI strains were calculated. A Student *t*-test was conducted, and the *P* value < 0.05 indicated a statistically significant difference.

## Supplementary Information

**Additional file 1:** **Table S1.** The primers used for RT-qPCR analysis.

**Additional file 2:** **Table S2.** The list of the differentially expressed genes

**Additional file 3:** **Figure** **S1.** The PCA plot by ggplot2. The △abaI mutant and wild type (wt) were clustered into two groups based on the gene expression matrix. **Figure S2.** Heatmap of the Pearson correlation coefficient between the samples by corrplot. **Figure S3.** The map of the enriched KEGG pathways from the KEGG database[1, 2]. Green rectangles represented the downregulated genes and red rectangles represented the upregulated genes. Blue boxes represented the genes hyperlinked to KO entries, and white boxes represented the genes which were not hyperlinked to KO entries. (A) Phenylalanine metabolism (ko00360). (B) The type VI secretion system of bacterial secretion system (ko03070). **Figure S4.** The map of the enriched KEGG pathway “propanoate metabolism (ko00640)” from the KEGG database[1, 2]. Green rectangles represented the downregulated genes and red rectangles represented the upregulated genes. Blue boxes represented the genes hyperlinked to KO entries, and white boxes represented the genes which were not hyperlinked to KO entries.

## Data Availability

The datasets presented in this study are available in NCBI Sequence Read Archive (SRA) with the accession number PRJNA770023.
