# Supplementary material for: Transcriptomic analysis reveals the regulatory role of quorum sensing in the Acinetobacter baumannii ATCC 19606 via RNA-seq
Source: BMC Microbiol. 2022 Aug 16;22:198. doi: 10.1186/s12866-022-02612-z (PMC9380347; doi:10.1186/s12866-022-02612-z)
Supplement: Supplementary file 1 — Additional file 1: Table S1. The primers used for RT-qPCR analysis. [file 12866_2022_2612_MOESM1_ESM.docx]

**Additional file 1**

**Table S1**. The primers used for RT-qPCR analysis

| **Target Gene** | **Forward primers sequence 5’ to 3’** | **Reverse primers sequence 5’ to 3’** |
| --- | --- | --- |
| FQU82_00189 | AATAGCGGTGTTGAGCAAGG | GGCACCCACAAAGTTACCTG |
| FQU82_00190 | GTTGCTGGAGTTCGGATGTA | CTGAGTCGCAAACCCATCTT |
| FQU82_01642 | GCAGGCTTAACGCTAGAACG | TACGACGGCTTACGCTTTTC |
| FQU82_00191 | TCAGCAGGTTTTCCAAGTCC | TGCTAAAATTCCCGGTGTGT |
| FQU82_01257 | GAGCTGGACACCAAAAATTCTGAT | CCGAAAATCCCACCAATACTAATTA |
| FQU82_01580 | TTGACTGGCACGAATTTAATGAA | TGCTTCGATACGCTCGTGAT |
| FQU82_01589 | TCGTGATTTGACCCGCTTACT | ACGACCCACAATTTTATCCATTTT |
| FQU82_00307 | TTAATCAGCAAGCACAACAGC | CTTGTACGATTGCAGCGAGAG |
| FQU82_00364 | GGGCAACAGCATTTATTCCTT | AGCATCAGCAGGTTGGAAAT |
| FQU82_02258 | GTCAGCCGTGGTCTATTGGT | AGAATTGAGCCCCACACATC |
| FQU82_01550 | ATATTGGGTGGGAAGTCTCG | ATCAACTCGTGGGTCTGCAT |
| FQU82_02259 | GCTTATGCTACGCACAGACG | GCCCCAATTACAAGGAAACA |
| FQU82_02260 | ATGTGGTATTTCGCATGGATTCT | GCTCAATACACCCGCGAAAC |
| FQU82_01549 | CGTATCAGACCAGCCTATGTTTCA | TTTCTTGCAGCACTTTCATTGC |
| FQU82_02635 | GAATGCCCCAATACGGCTA | TTGGACAAACCGCCTTACA |
