# Supplementary material for: Transcriptomic analysis reveals the regulatory role of quorum sensing in the Acinetobacter baumannii ATCC 19606 via RNA-seq
Source: BMC Microbiol. 2022 Aug 16;22:198. doi: 10.1186/s12866-022-02612-z (PMC9380347; doi:10.1186/s12866-022-02612-z)
Supplement: Supplementary file 2 — Additional file 2: Table S2. The list of the differentially expressed genes [file 12866_2022_2612_MOESM2_ESM.docx]

**Additional file 2**

**Table S2.** The list of the differentially expressed genes

| **gene_id** | **baseMean** | **log2FoldChange** | **pvalue** | **padj** |
| --- | --- | --- | --- | --- |
| FQU82_00195 | 191.7724336 | 2.157166461 | 5.95E-38 | 2.04E-34 |
| FQU82_00072 | 2283.169192 | 0.944273111 | 5.56E-28 | 9.52E-25 |
| FQU82_01211 | 1914.368011 | -1.134306169 | 2.17E-27 | 2.47E-24 |
| FQU82_00071 | 1656.981251 | 0.928718294 | 1.01E-26 | 8.62E-24 |
| FQU82_00221 | 2548.442001 | 1.080326188 | 8.16E-23 | 5.59E-20 |
| FQU82_00616 | 576.690735 | -0.986938191 | 1.81E-21 | 1.03E-18 |
| FQU82_03539 | 1100.563988 | 0.802870619 | 1.02E-20 | 4.98E-18 |
| FQU82_00188 | 432.0826459 | 1.517427381 | 1.34E-20 | 5.72E-18 |
| FQU82_00216 | 2431.112888 | 0.872208855 | 3.49E-19 | 1.33E-16 |
| FQU82_02188 | 126.0802593 | 1.479948703 | 6.70E-19 | 2.29E-16 |
| FQU82_00069 | 849.5058836 | 0.925206376 | 2.04E-18 | 6.34E-16 |
| FQU82_01257 | 136.9482794 | 1.578761659 | 1.35E-17 | 3.84E-15 |
| FQU82_00197 | 65.10827781 | 2.280061809 | 1.81E-17 | 4.43E-15 |
| FQU82_00222 | 453.0239475 | 0.939886051 | 1.72E-17 | 4.43E-15 |
| FQU82_00187 | 5663.298525 | 1.041463982 | 3.19E-17 | 7.28E-15 |
| FQU82_00205 | 603.2953777 | 1.275605881 | 8.34E-17 | 1.78E-14 |
| FQU82_00242 | 1020.969127 | 0.870355645 | 1.10E-16 | 2.22E-14 |
| FQU82_00054 | 1426.823599 | 0.947595936 | 2.52E-16 | 4.79E-14 |
| FQU82_00164 | 149.9903732 | 1.420267937 | 5.44E-16 | 9.80E-14 |
| FQU82_00202 | 680.1670111 | 0.972018935 | 7.12E-16 | 1.22E-13 |
| FQU82_00132 | 4607.172037 | 0.71569486 | 1.05E-15 | 1.72E-13 |
| FQU82_00165 | 289.9708207 | 1.095250322 | 4.73E-15 | 7.36E-13 |
| FQU82_01536 | 5895.9607 | -0.882181928 | 7.12E-15 | 1.06E-12 |
| FQU82_00186 | 6165.490206 | 1.213934659 | 6.89E-14 | 9.83E-12 |
| FQU82_01545 | 1470.473884 | -0.963241061 | 9.21E-14 | 1.26E-11 |
| FQU82_00070 | 5137.045246 | 0.875235006 | 1.27E-13 | 1.67E-11 |
| FQU82_00243 | 1226.940152 | 0.628680895 | 1.38E-13 | 1.75E-11 |
| FQU82_00131 | 998.5290743 | 0.758165713 | 1.58E-13 | 1.93E-11 |
| FQU82_00211 | 494.5441449 | 0.807903583 | 3.02E-13 | 3.56E-11 |
| FQU82_00204 | 403.891313 | 1.084025761 | 3.34E-13 | 3.81E-11 |
| FQU82_01550 | 2917.119026 | -1.082900348 | 4.15E-13 | 4.58E-11 |
| FQU82_00157 | 19143.40366 | 0.892695244 | 4.55E-13 | 4.79E-11 |
| FQU82_00644 | 545.9372385 | 0.960880862 | 4.62E-13 | 4.79E-11 |
| FQU82_02174 | 389.6976238 | 1.025939964 | 7.66E-13 | 7.71E-11 |
| FQU82_00214 | 685.1656151 | 0.82453941 | 9.86E-13 | 9.37E-11 |
| FQU82_01537 | 2867.202257 | -0.782909309 | 9.84E-13 | 9.37E-11 |
| FQU82_00074 | 563.8137724 | 1.052319804 | 2.51E-12 | 2.32E-10 |
| FQU82_00203 | 644.8194063 | 0.889540034 | 2.80E-12 | 2.52E-10 |
| FQU82_00246 | 374.7676426 | 1.029162756 | 2.91E-12 | 2.55E-10 |
| FQU82_00149 | 7634.078426 | 0.608463347 | 3.09E-12 | 2.64E-10 |
| FQU82_00073 | 2826.479919 | 0.685737625 | 4.87E-12 | 4.06E-10 |
| FQU82_02421 | 993.9200125 | 0.952187758 | 5.09E-12 | 4.14E-10 |
| FQU82_00591 | 4224.241686 | -0.638930502 | 5.33E-12 | 4.24E-10 |
| FQU82_00194 | 478.6643185 | 0.938742266 | 5.60E-12 | 4.35E-10 |
| FQU82_01549 | 2161.935204 | -0.982651829 | 5.73E-12 | 4.35E-10 |
| FQU82_03254 | 740.8299511 | -0.803733958 | 6.73E-12 | 5.01E-10 |
| FQU82_00184 | 2795.986427 | 1.506123822 | 7.19E-12 | 5.23E-10 |
| FQU82_01538 | 25844.8163 | -0.804624727 | 7.46E-12 | 5.32E-10 |
| FQU82_00220 | 189.7945963 | 0.996652603 | 8.36E-12 | 5.72E-10 |
| FQU82_00158 | 628.3084498 | 1.067968054 | 2.48E-11 | 1.63E-09 |
| FQU82_01544 | 7746.157347 | -0.886208861 | 4.70E-11 | 3.04E-09 |
| FQU82_00169 | 229.8963785 | 1.383977711 | 4.85E-11 | 3.08E-09 |
| FQU82_00198 | 54.46049869 | 1.506257099 | 5.42E-11 | 3.37E-09 |
| FQU82_03538 | 472.6210542 | 0.874187447 | 5.86E-11 | 3.58E-09 |
| FQU82_00190 | 375.7415656 | 1.843594526 | 1.16E-10 | 6.82E-09 |
| FQU82_01546 | 1553.565208 | -0.919491695 | 1.15E-10 | 6.82E-09 |
| FQU82_00061 | 958.1676108 | 0.664634966 | 1.44E-10 | 8.38E-09 |
| FQU82_00191 | 290.0067069 | 1.826250534 | 1.97E-10 | 1.11E-08 |
| FQU82_00067 | 681.6406003 | 0.660132703 | 2.19E-10 | 1.21E-08 |
| FQU82_01552 | 714.3753007 | -0.838667937 | 2.31E-10 | 1.26E-08 |
| FQU82_01548 | 5007.572787 | -0.988601021 | 2.48E-10 | 1.32E-08 |
| FQU82_03533 | 607.398682 | 0.761119255 | 3.32E-10 | 1.72E-08 |
| FQU82_01982 | 931.2758896 | 1.288518397 | 3.47E-10 | 1.77E-08 |
| FQU82_00210 | 891.1535633 | 0.836228714 | 4.50E-10 | 2.26E-08 |
| FQU82_00199 | 71.1379467 | 1.464351861 | 4.80E-10 | 2.35E-08 |
| FQU82_00307 | 503.0337459 | 0.668878059 | 4.76E-10 | 2.35E-08 |
| FQU82_00956 | 976.7056993 | -0.641760915 | 5.51E-10 | 2.66E-08 |
| FQU82_03692 | 1008.035397 | -0.719239154 | 5.69E-10 | 2.71E-08 |
| FQU82_00213 | 623.8773503 | 0.620414875 | 6.67E-10 | 3.13E-08 |
| FQU82_01551 | 1170.961681 | -0.900934741 | 7.25E-10 | 3.31E-08 |
| FQU82_00247 | 502.3202348 | 0.782754429 | 1.09E-09 | 4.90E-08 |
| FQU82_01540 | 1963.505841 | -0.743685963 | 1.13E-09 | 5.02E-08 |
| FQU82_00223 | 528.9390783 | 0.732431574 | 1.15E-09 | 5.02E-08 |
| FQU82_00183 | 6036.45071 | 1.158651006 | 1.33E-09 | 5.74E-08 |
| FQU82_00228 | 302.9438193 | 0.656822862 | 1.49E-09 | 6.37E-08 |
| FQU82_00521 | 595.4593902 | -0.71919991 | 1.83E-09 | 7.73E-08 |
| FQU82_01539 | 22822.46226 | -0.978871405 | 1.91E-09 | 7.97E-08 |
| FQU82_01553 | 772.0863163 | -0.840110585 | 2.22E-09 | 9.14E-08 |
| FQU82_00952 | 1171.298324 | -0.697129324 | 2.28E-09 | 9.27E-08 |
| FQU82_03544 | 2163.712022 | -0.667722262 | 2.85E-09 | 1.15E-07 |
| FQU82_01900 | 384.8215983 | 0.861874422 | 3.66E-09 | 1.44E-07 |
| FQU82_00185 | 2099.678258 | 1.059169643 | 4.49E-09 | 1.75E-07 |
| FQU82_01003 | 243.5520247 | 0.934423238 | 4.86E-09 | 1.87E-07 |
| FQU82_00212 | 682.0239768 | 0.781091186 | 5.60E-09 | 2.13E-07 |
| FQU82_00562 | 4213.042707 | 1.1632434 | 6.33E-09 | 2.36E-07 |
| FQU82_00250 | 686.3146445 | 0.691292424 | 6.55E-09 | 2.41E-07 |
| FQU82_03303 | 846.1984706 | -0.767961326 | 7.53E-09 | 2.74E-07 |
| FQU82_00248 | 415.0687652 | 0.726191991 | 7.63E-09 | 2.75E-07 |
| FQU82_00126 | 204.4972105 | 0.974785316 | 8.87E-09 | 3.16E-07 |
| FQU82_00179 | 239.3663371 | 1.026002271 | 9.64E-09 | 3.37E-07 |
| FQU82_00209 | 764.2208488 | 0.703847351 | 1.03E-08 | 3.57E-07 |
| FQU82_00910 | 2881.40106 | 0.737557581 | 1.34E-08 | 4.53E-07 |
| FQU82_01653 | 381.610102 | 0.70915152 | 1.61E-08 | 5.39E-07 |
| FQU82_02035 | 91.07781885 | 1.044653164 | 1.80E-08 | 5.98E-07 |
| FQU82_02739 | 1362.833778 | 0.858719795 | 2.30E-08 | 7.56E-07 |
| FQU82_00953 | 313.0814656 | -0.91498203 | 2.37E-08 | 7.67E-07 |
| FQU82_03369 | 2195.364972 | 0.778855126 | 2.64E-08 | 8.38E-07 |
| FQU82_00059 | 93.22823169 | 1.26410035 | 3.04E-08 | 9.56E-07 |
| FQU82_02902 | 402.804253 | -0.793681577 | 4.32E-08 | 1.33E-06 |
| FQU82_00162 | 395.1968208 | 0.894311285 | 4.78E-08 | 1.45E-06 |
| FQU82_01633 | 237.848248 | 0.858406565 | 4.90E-08 | 1.47E-06 |
| FQU82_01547 | 426.2215228 | -0.924495713 | 5.62E-08 | 1.67E-06 |
| FQU82_00364 | 5984.805739 | -1.195721702 | 6.27E-08 | 1.85E-06 |
| FQU82_01133 | 1168.227701 | -0.6669299 | 7.30E-08 | 2.14E-06 |
| FQU82_02646 | 214.9288202 | 0.954606295 | 9.67E-08 | 2.81E-06 |
| FQU82_00219 | 91.60823753 | 1.030283515 | 9.87E-08 | 2.84E-06 |
| FQU82_02324 | 1032.61247 | 0.813369807 | 1.01E-07 | 2.87E-06 |
| FQU82_03627 | 1499.271375 | 1.16214768 | 1.06E-07 | 3.01E-06 |
| FQU82_03626 | 13235.73775 | 1.091211719 | 1.12E-07 | 3.15E-06 |
| FQU82_03075 | 1221.079908 | -1.009229527 | 1.28E-07 | 3.55E-06 |
| FQU82_00065 | 91.90071653 | 1.045402892 | 1.48E-07 | 4.08E-06 |
| FQU82_00747 | 283.1629483 | 0.70441685 | 1.50E-07 | 4.08E-06 |
| FQU82_03116 | 196.3146132 | 0.714048989 | 1.50E-07 | 4.08E-06 |
| FQU82_00163 | 311.0179261 | 0.985144709 | 1.64E-07 | 4.32E-06 |
| FQU82_00950 | 160.9314845 | 0.797658918 | 1.62E-07 | 4.32E-06 |
| FQU82_02222 | 175.6934211 | 0.909623028 | 1.64E-07 | 4.32E-06 |
| FQU82_00064 | 121.1186232 | 1.134635137 | 1.73E-07 | 4.50E-06 |
| FQU82_02258 | 20924.77494 | -1.103052361 | 1.87E-07 | 4.82E-06 |
| FQU82_00206 | 103.5892906 | 1.659297553 | 1.90E-07 | 4.85E-06 |
| FQU82_03635 | 3651.446144 | 0.935384758 | 2.22E-07 | 5.62E-06 |
| FQU82_01241 | 1100.55696 | 0.615029524 | 2.36E-07 | 5.93E-06 |
| FQU82_02259 | 13060.25174 | -1.07321689 | 2.38E-07 | 5.95E-06 |
| FQU82_00249 | 295.610192 | 0.690861723 | 2.56E-07 | 6.36E-06 |
| FQU82_00141 | 680.6601273 | 0.619735439 | 2.67E-07 | 6.57E-06 |
| FQU82_01225 | 1217.750992 | 1.082859078 | 2.80E-07 | 6.84E-06 |
| FQU82_01753 | 8938.297564 | 1.258869454 | 3.53E-07 | 8.56E-06 |
| FQU82_03018 | 1900.200844 | -0.738507951 | 4.00E-07 | 9.58E-06 |
| FQU82_02171 | 45.59948246 | 1.263514502 | 4.50E-07 | 1.06E-05 |
| FQU82_01642 | 24.79280116 | 1.841727283 | 5.60E-07 | 1.29E-05 |
| FQU82_00783 | 5619.904195 | -0.609039665 | 5.74E-07 | 1.32E-05 |
| FQU82_00180 | 303.6015241 | 0.606744111 | 6.68E-07 | 1.50E-05 |
| FQU82_02260 | 294.0290672 | -1.067787104 | 6.78E-07 | 1.52E-05 |
| FQU82_00490 | 2864.597895 | -0.770300113 | 6.86E-07 | 1.53E-05 |
| FQU82_01242 | 2041.484561 | 0.622459563 | 7.34E-07 | 1.62E-05 |
| FQU82_00168 | 43.43447474 | 1.499678118 | 7.53E-07 | 1.65E-05 |
| FQU82_02018 | 535.350657 | 0.792837821 | 7.82E-07 | 1.70E-05 |
| FQU82_01341 | 7739.08857 | -0.765253488 | 8.41E-07 | 1.82E-05 |
| FQU82_01849 | 3799.397554 | -0.750284766 | 9.18E-07 | 1.98E-05 |
| FQU82_01280 | 9936.395075 | -0.728466675 | 9.31E-07 | 1.99E-05 |
| FQU82_00178 | 217.6445311 | 0.960604724 | 9.90E-07 | 2.10E-05 |
| FQU82_00607 | 2563.631614 | -0.738028168 | 9.92E-07 | 2.10E-05 |
| FQU82_00192 | 307.1075782 | 0.879130239 | 1.06E-06 | 2.22E-05 |
| FQU82_01785 | 313.8279005 | -0.661390651 | 1.10E-06 | 2.28E-05 |
| FQU82_00129 | 197.6863094 | 0.719575384 | 1.20E-06 | 2.47E-05 |
| FQU82_01008 | 3888.140876 | -0.779584999 | 1.42E-06 | 2.90E-05 |
| FQU82_02633 | 176.8591262 | 0.812050696 | 1.46E-06 | 2.97E-05 |
| FQU82_01177 | 11111.58491 | -0.757275841 | 1.62E-06 | 3.23E-05 |
| FQU82_02503 | 8790.972814 | -0.691956605 | 1.61E-06 | 3.23E-05 |
| FQU82_00062 | 72.30567952 | 1.009793648 | 1.64E-06 | 3.27E-05 |
| FQU82_00215 | 123.4064817 | 1.069803793 | 1.72E-06 | 3.41E-05 |
| FQU82_02218 | 196.2085378 | 0.736584831 | 1.75E-06 | 3.45E-05 |
| FQU82_01357 | 2244.364233 | -0.749977398 | 2.01E-06 | 3.92E-05 |
| FQU82_00576 | 20356.93184 | -0.670579671 | 2.25E-06 | 4.33E-05 |
| FQU82_01937 | 1190.448967 | -0.640848624 | 2.45E-06 | 4.66E-05 |
| FQU82_03368 | 458.7269184 | 0.906892074 | 2.44E-06 | 4.66E-05 |
| FQU82_02944 | 20300.14406 | -0.672924335 | 2.57E-06 | 4.86E-05 |
| FQU82_00167 | 99.77085907 | 0.842228311 | 2.92E-06 | 5.43E-05 |
| FQU82_01541 | 2415.402027 | -0.690175088 | 2.94E-06 | 5.45E-05 |
| FQU82_00608 | 266.3670628 | -0.591350749 | 3.40E-06 | 6.25E-05 |
| FQU82_03541 | 1445.459651 | 0.708379499 | 3.51E-06 | 6.42E-05 |
| FQU82_01845 | 477.8014369 | -0.72812216 | 3.54E-06 | 6.45E-05 |
| FQU82_01239 | 130.7266617 | 0.996641893 | 3.75E-06 | 6.76E-05 |
| FQU82_01217 | 7199.861141 | -0.825984354 | 3.93E-06 | 6.99E-05 |
| FQU82_01543 | 1410.503756 | -0.709948802 | 3.96E-06 | 6.99E-05 |
| FQU82_02187 | 165.6081242 | 1.066318917 | 3.95E-06 | 6.99E-05 |
| FQU82_03188 | 1939.898493 | 0.656342127 | 3.96E-06 | 6.99E-05 |
| FQU82_02028 | 117.9408319 | 0.991862904 | 4.33E-06 | 7.53E-05 |
| FQU82_02508 | 16038.68065 | -0.613409029 | 4.37E-06 | 7.55E-05 |
| FQU82_01034 | 6419.527069 | -0.745825293 | 4.42E-06 | 7.60E-05 |
| FQU82_00189 | 1064.377569 | 1.971596606 | 4.78E-06 | 8.10E-05 |
| FQU82_01728 | 191.5362802 | -0.798993998 | 4.83E-06 | 8.13E-05 |
| FQU82_01823 | 319.9209762 | 0.628814622 | 4.92E-06 | 8.21E-05 |
| FQU82_03233 | 240.1649095 | 0.613389839 | 5.12E-06 | 8.50E-05 |
| FQU82_03037 | 2175.843002 | -0.658121218 | 5.91E-06 | 9.63E-05 |
| FQU82_01224 | 686.6957625 | 0.741972045 | 6.00E-06 | 9.72E-05 |
| FQU82_00193 | 227.7783802 | 1.064317138 | 6.23E-06 | 0.00010052 |
| FQU82_02137 | 64.77047092 | 0.891654045 | 6.40E-06 | 0.000102846 |
| FQU82_00948 | 191.8885479 | 0.650616313 | 6.87E-06 | 0.000109886 |
| FQU82_01255 | 270.5591446 | 0.72966531 | 7.48E-06 | 0.000118473 |
| FQU82_01779 | 1549.315821 | 0.858742835 | 7.72E-06 | 0.000121751 |
| FQU82_03775 | 13191.397 | 0.674095693 | 8.16E-06 | 0.000128076 |
| FQU82_03198 | 1995.757717 | -0.773754069 | 8.91E-06 | 0.000137931 |
| FQU82_00996 | 701.851315 | 0.601056829 | 9.54E-06 | 0.000147029 |
| FQU82_02017 | 598.106066 | 0.86249107 | 9.81E-06 | 0.000149261 |
| FQU82_01769 | 4372.917986 | -0.859501533 | 1.01E-05 | 0.000152352 |
| FQU82_02813 | 7581.305277 | 0.626140197 | 1.05E-05 | 0.000157027 |
| FQU82_00060 | 36.35188151 | 1.359303659 | 1.17E-05 | 0.000171466 |
| FQU82_02261 | 1358.852111 | -0.891491231 | 1.17E-05 | 0.000171466 |
| FQU82_01215 | 3633.661115 | -0.841787975 | 1.18E-05 | 0.000172633 |
| FQU82_01216 | 3553.206079 | -0.80355235 | 1.23E-05 | 0.000178187 |
| FQU82_01365 | 348.1140078 | -0.732945118 | 1.28E-05 | 0.000184538 |
| FQU82_01578 | 613.8918757 | 0.76180542 | 1.31E-05 | 0.000187163 |
| FQU82_01355 | 5225.451485 | -0.603269334 | 1.32E-05 | 0.000188034 |
| FQU82_00159 | 1461.929438 | 0.907453092 | 1.37E-05 | 0.000193499 |
| FQU82_02474 | 1781.26259 | -0.630117897 | 1.37E-05 | 0.000193499 |
| FQU82_03063 | 2813.191838 | -0.604341884 | 1.72E-05 | 0.00023774 |
| FQU82_00363 | 2979.793106 | -0.672462194 | 1.92E-05 | 0.000262788 |
| FQU82_02026 | 899.7971705 | 0.663851579 | 2.41E-05 | 0.000324168 |
| FQU82_00160 | 2619.12801 | 0.968414446 | 2.47E-05 | 0.00032652 |
| FQU82_01256 | 247.9894986 | 0.754220068 | 2.47E-05 | 0.00032652 |
| FQU82_00984 | 96.47752581 | 0.879428873 | 2.51E-05 | 0.000330188 |
| FQU82_01778 | 3421.561686 | 0.838412506 | 2.71E-05 | 0.000352071 |
| FQU82_02223 | 209.1346663 | 0.893572072 | 2.74E-05 | 0.000354793 |
| FQU82_02473 | 599.8323042 | -0.599148295 | 2.84E-05 | 0.000365377 |
| FQU82_02766 | 2449.011664 | 0.767750233 | 2.87E-05 | 0.000367433 |
| FQU82_00939 | 793.0384305 | -0.638826465 | 2.96E-05 | 0.000374946 |
| FQU82_00466 | 1152.183707 | -0.815761496 | 3.19E-05 | 0.000401862 |
| FQU82_02957 | 648.2999375 | -0.629534929 | 3.46E-05 | 0.000432092 |
| FQU82_00941 | 504.5874068 | -0.60134638 | 3.50E-05 | 0.000433637 |
| FQU82_01249 | 132.1298942 | 0.718621838 | 3.52E-05 | 0.000434602 |
| FQU82_03197 | 47880.32657 | -0.59246461 | 3.56E-05 | 0.000438819 |
| FQU82_00201 | 33.58699845 | 1.169697526 | 3.66E-05 | 0.000447634 |
| FQU82_00066 | 65.2099772 | 1.080440758 | 3.80E-05 | 0.000460645 |
| FQU82_01258 | 26.90587455 | 1.203397305 | 3.84E-05 | 0.000462217 |
| FQU82_00182 | 56.83820215 | 0.989813535 | 3.93E-05 | 0.000470119 |
| FQU82_03530 | 402.4404201 | 0.714519686 | 4.01E-05 | 0.000476378 |
| FQU82_02213 | 143.6786918 | 0.819926644 | 4.15E-05 | 0.000489709 |
| FQU82_02190 | 26.13837694 | 1.269952915 | 4.38E-05 | 0.000513253 |
| FQU82_01666 | 29.28139893 | -1.762318703 | 4.60E-05 | 0.00053712 |
| FQU82_03750 | 328.933515 | 0.65265292 | 4.90E-05 | 0.000566117 |
| FQU82_02257 | 55.3832983 | -1.325980427 | 5.14E-05 | 0.00058595 |
| FQU82_00200 | 131.8307595 | 0.864848504 | 5.22E-05 | 0.000593853 |
| FQU82_00721 | 118.8557789 | 0.800097624 | 5.77E-05 | 0.000635733 |
| FQU82_00806 | 368.1851192 | -0.689083544 | 5.78E-05 | 0.000635733 |
| FQU82_01274 | 4354.087563 | -0.646232914 | 5.83E-05 | 0.000639435 |
| FQU82_01824 | 6802.735704 | -0.591908251 | 5.88E-05 | 0.000641729 |
| FQU82_02214 | 76.57521213 | 0.749506743 | 5.89E-05 | 0.000641729 |
| FQU82_02575 | 170.0121742 | 0.716679819 | 6.70E-05 | 0.000716946 |
| FQU82_03566 | 102.5802155 | 0.757331352 | 6.79E-05 | 0.000723819 |
| FQU82_02221 | 416.4150792 | 0.661070587 | 7.17E-05 | 0.000759648 |
| FQU82_03466 | 114.7294162 | 0.706576512 | 7.17E-05 | 0.000759648 |
| FQU82_02901 | 8999.884672 | -0.649586973 | 7.23E-05 | 0.00076206 |
| FQU82_01542 | 882.564349 | -0.636208224 | 7.30E-05 | 0.000766435 |
| FQU82_00853 | 216.667802 | 0.601609504 | 7.40E-05 | 0.00077488 |
| FQU82_00986 | 37.13252747 | 1.22000262 | 7.51E-05 | 0.000783984 |
| FQU82_03199 | 2035.491647 | -0.633893539 | 7.60E-05 | 0.000790944 |
| FQU82_00036 | 4039.926234 | -0.596529897 | 7.80E-05 | 0.000800109 |
| FQU82_00218 | 100.5320625 | 0.739804653 | 8.77E-05 | 0.000879786 |
| FQU82_01052 | 291.5519353 | -0.873280258 | 9.07E-05 | 0.000901857 |
| FQU82_03323 | 98.19449391 | 0.614725683 | 9.84E-05 | 0.000972962 |
| FQU82_03324 | 208.5361807 | 0.674491597 | 0.000110692 | 0.001079171 |
| FQU82_01710 | 99.42948594 | 0.856620395 | 0.000112294 | 0.001091679 |
| FQU82_02315 | 1922.248444 | -0.591046425 | 0.000133333 | 0.001271761 |
| FQU82_02126 | 38.8515123 | 0.942608487 | 0.000137322 | 0.001301704 |
| FQU82_02019 | 188.1486129 | 0.6409803 | 0.000140974 | 0.001332636 |
| FQU82_00970 | 983.4734448 | 0.635461806 | 0.00014585 | 0.001363655 |
| FQU82_01780 | 1638.361927 | 0.766239124 | 0.000148345 | 0.00138321 |
| FQU82_00716 | 1810.089517 | -0.609872047 | 0.000149766 | 0.001392664 |
| FQU82_00779 | 749.4944635 | 0.630366827 | 0.000150927 | 0.001395869 |
| FQU82_01050 | 324.4139192 | -0.646427352 | 0.000150885 | 0.001395869 |
| FQU82_00161 | 5620.8073 | 0.821561832 | 0.000164397 | 0.001492216 |
| FQU82_03615 | 1553.835484 | -0.670328063 | 0.000173023 | 0.001554024 |
| FQU82_02645 | 20943.14634 | -0.598365181 | 0.000176459 | 0.001572508 |
| FQU82_02833 | 8297.534153 | -0.600254646 | 0.000183696 | 0.001624312 |
| FQU82_00006 | 3939.813927 | 0.611682686 | 0.000184547 | 0.001627627 |
| FQU82_02212 | 108.8116979 | 0.676376995 | 0.000199272 | 0.001735139 |
| FQU82_00717 | 210.2113494 | -0.733483276 | 0.000229995 | 0.001957815 |
| FQU82_01881 | 821.7646172 | -0.631947391 | 0.0002345 | 0.001991215 |
| FQU82_00795 | 311.5995245 | -0.643171591 | 0.000272821 | 0.002266006 |
| FQU82_02872 | 51008.15502 | -0.609081062 | 0.000302626 | 0.002465677 |
| FQU82_00176 | 44.36938817 | 0.998028596 | 0.000307397 | 0.002494593 |
| FQU82_02172 | 66.12432846 | 1.072340928 | 0.00031284 | 0.002524859 |
| FQU82_01978 | 2143.439611 | -0.59518313 | 0.000339429 | 0.002726585 |
| FQU82_00063 | 54.11594309 | 0.87520173 | 0.000359241 | 0.002852256 |
| FQU82_00985 | 37.52524024 | 1.163222979 | 0.000361849 | 0.002859694 |
| FQU82_01752 | 9511.894854 | 0.688626632 | 0.000363291 | 0.002862181 |
| FQU82_01383 | 331.416379 | 0.718591158 | 0.000365353 | 0.002867521 |
| FQU82_01053 | 54.96369668 | -0.81262739 | 0.000392036 | 0.003021502 |
| FQU82_00723 | 73.69141538 | 0.869227981 | 0.000392959 | 0.003021807 |
| FQU82_02770 | 138.341701 | 0.643061947 | 0.00040445 | 0.003082466 |
| FQU82_02150 | 86.20138758 | 0.718330972 | 0.000406666 | 0.003092466 |
| FQU82_02507 | 3654.407454 | -0.607782128 | 0.000414579 | 0.003131766 |
| FQU82_02293 | 99.1037847 | 0.810090538 | 0.000424738 | 0.0031876 |
| FQU82_02634 | 819.0551022 | -0.808697552 | 0.00043156 | 0.003217426 |
| FQU82_03629 | 2089.513939 | 0.668656801 | 0.000455422 | 0.003380596 |
| FQU82_02840 | 451.6555789 | -0.614920517 | 0.000494262 | 0.003592789 |
| FQU82_00245 | 1319.851654 | 0.88709176 | 0.000509883 | 0.003681053 |
| FQU82_00719 | 699.7629304 | 0.726786017 | 0.000521481 | 0.003719325 |
| FQU82_03050 | 10476.97061 | -0.589150518 | 0.000529906 | 0.003769935 |
| FQU82_00652 | 85.59099898 | -0.740731929 | 0.000624361 | 0.004333798 |
| FQU82_02544 | 45.16698569 | 0.774540133 | 0.000643315 | 0.004438352 |
| FQU82_00464 | 161.9111 | 0.66931698 | 0.000677113 | 0.004652776 |
| FQU82_00154 | 2478.779299 | 0.925769795 | 0.000688347 | 0.00472049 |
| FQU82_02461 | 144.8989414 | 0.998512471 | 0.00073946 | 0.005000037 |
| FQU82_00153 | 12599.21092 | 0.779457468 | 0.000755201 | 0.005057335 |
| FQU82_01994 | 132.0914659 | -0.624160168 | 0.000811763 | 0.005342027 |
| FQU82_02572 | 126.1068547 | 0.728639818 | 0.000885777 | 0.005719113 |
| FQU82_02748 | 16774.44269 | -0.604160674 | 0.000902798 | 0.005785346 |
| FQU82_03444 | 91.27603567 | 0.640960097 | 0.000901482 | 0.005785346 |
| FQU82_01604 | 105.8235651 | 0.594698631 | 0.000940688 | 0.005917342 |
| FQU82_03604 | 102.4657805 | 1.103822448 | 0.000947712 | 0.005950586 |
| FQU82_03477 | 75.2501732 | 0.847099374 | 0.000978912 | 0.006112839 |
| FQU82_01051 | 136.1637497 | -0.803789988 | 0.001006528 | 0.006262435 |
| FQU82_00995 | 55.37230908 | 0.837879087 | 0.001121069 | 0.006850534 |
| FQU82_00244 | 138.7554759 | 1.145294851 | 0.001251858 | 0.007542001 |
| FQU82_02294 | 128.981708 | 0.745728439 | 0.001250811 | 0.007542001 |
| FQU82_01952 | 69.4201692 | -0.628874395 | 0.001325019 | 0.007885594 |
| FQU82_02385 | 23.13303112 | -1.044381911 | 0.001435874 | 0.008428062 |
| FQU82_02456 | 68.05061864 | 0.597932384 | 0.00146945 | 0.008566367 |
| FQU82_03734 | 59.96959039 | -0.62360028 | 0.001531549 | 0.008838045 |
| FQU82_02460 | 164.2401659 | 0.858104292 | 0.001597355 | 0.009156028 |
| FQU82_02351 | 128.6132042 | 0.742547962 | 0.001665929 | 0.009485537 |
| FQU82_01636 | 71.1359445 | 0.640134439 | 0.00170002 | 0.009631573 |
| FQU82_00254 | 20.25401302 | 1.100743694 | 0.001753055 | 0.009915626 |
| FQU82_00068 | 38.45839164 | 1.292703509 | 0.001779837 | 0.010017437 |
| FQU82_00947 | 31.72455763 | 0.928865193 | 0.001866873 | 0.010438625 |
| FQU82_00170 | 61.03734993 | 0.905568587 | 0.002347707 | 0.012455586 |
| FQU82_00370 | 74.59848227 | 0.645702338 | 0.002356691 | 0.012464602 |
| FQU82_01376 | 32.50995529 | 0.816076356 | 0.002410839 | 0.012672643 |
| FQU82_02515 | 2444.598083 | -0.639729429 | 0.002428112 | 0.012743864 |
| FQU82_00554 | 2013.596118 | -0.646346226 | 0.002581398 | 0.013363911 |
| FQU82_01716 | 62.00524606 | 0.631110201 | 0.002618633 | 0.013495423 |
| FQU82_01680 | 76.57286437 | 0.719031803 | 0.002892104 | 0.014603951 |
| FQU82_01589 | 305.6351064 | 0.67484781 | 0.002924108 | 0.014693535 |
| FQU82_01647 | 26.15888051 | 0.943291522 | 0.002947879 | 0.014781225 |
| FQU82_01797 | 133.3670869 | 0.5902021 | 0.00298864 | 0.014874247 |
| FQU82_00256 | 36.80442179 | 0.807643965 | 0.003040033 | 0.015098685 |
| FQU82_01616 | 29.3392284 | 0.912957063 | 0.003268598 | 0.015955981 |
| FQU82_00166 | 73.45885933 | 0.590674328 | 0.003347114 | 0.016292781 |
| FQU82_00456 | 41.30451257 | -0.726572446 | 0.003396254 | 0.016392073 |
| FQU82_03443 | 228.4057283 | 0.618555971 | 0.003458319 | 0.016668123 |
| FQU82_02184 | 63.28631755 | 0.61892738 | 0.00349339 | 0.016813475 |
| FQU82_01273 | 63.0057265 | -0.657131921 | 0.00364932 | 0.017392719 |
| FQU82_02905 | 293.746769 | 0.637502472 | 0.004365341 | 0.019838243 |
| FQU82_02805 | 97.80015742 | 0.612978132 | 0.004403264 | 0.019932697 |
| FQU82_01049 | 45.77531002 | -0.689102315 | 0.00444458 | 0.020058443 |
| FQU82_02758 | 14.1766057 | 1.309530713 | 0.004448965 | 0.020058443 |
| FQU82_03438 | 238.2684227 | 0.590494308 | 0.004712509 | 0.021025039 |
| FQU82_01467 | 16.86056119 | -1.006984662 | 0.004743952 | 0.021137765 |
| FQU82_00267 | 47.34248222 | 0.651916226 | 0.004763567 | 0.021170034 |
| FQU82_01644 | 62.30334062 | 0.62054049 | 0.004893271 | 0.02169012 |
| FQU82_00990 | 41.89678025 | 0.663086703 | 0.005216191 | 0.02259469 |
| FQU82_00102 | 88.72533699 | 0.992284481 | 0.00526256 | 0.022766728 |
| FQU82_02343 | 70.97295972 | 0.679741265 | 0.005296273 | 0.022854786 |
| FQU82_02635 | 72.26745157 | -0.729207824 | 0.005321342 | 0.022905198 |
| FQU82_02397 | 13.85769427 | 1.180239441 | 0.005343687 | 0.02295021 |
| FQU82_02011 | 12420.31967 | 0.700022184 | 0.005352988 | 0.022954477 |
| FQU82_01398 | 12.1537992 | 1.362092361 | 0.00540558 | 0.023012717 |
| FQU82_02842 | 121.4353219 | 0.997127031 | 0.005410571 | 0.023012717 |
| FQU82_03447 | 36.96213764 | 0.801011747 | 0.005692041 | 0.02395838 |
| FQU82_01226 | 87.59511963 | 0.590384348 | 0.005738102 | 0.024070117 |
| FQU82_01674 | 2201.846523 | -0.748128975 | 0.005743602 | 0.024070117 |
| FQU82_02143 | 9.642257166 | 1.439541907 | 0.006594741 | 0.02670675 |
| FQU82_01641 | 29.29050352 | 0.869351728 | 0.006612185 | 0.02670783 |
| FQU82_01970 | 78.03290103 | -0.681984867 | 0.006618422 | 0.02670783 |
| FQU82_03529 | 5239.777488 | 0.592627502 | 0.006772749 | 0.02713858 |
| FQU82_00545 | 55.91361971 | -0.595458718 | 0.006957416 | 0.027787652 |
| FQU82_01581 | 202.6318728 | 0.887506203 | 0.007317386 | 0.028981593 |
| FQU82_01580 | 643.9856328 | 1.218030354 | 0.007687909 | 0.030100715 |
| FQU82_01588 | 287.4143307 | 0.625773503 | 0.007745013 | 0.030220565 |
| FQU82_03743 | 40.7849966 | 0.689460152 | 0.007886252 | 0.030736624 |
| FQU82_03741 | 25.82459651 | 0.771373468 | 0.008097248 | 0.031238764 |
| FQU82_01571 | 36.30368052 | 0.800727587 | 0.008246319 | 0.031706633 |
| FQU82_01938 | 14.68670583 | -1.012531999 | 0.008316335 | 0.031793122 |
| FQU82_02170 | 32.05276777 | 0.831279939 | 0.008464512 | 0.032112594 |
| FQU82_01717 | 39.06544216 | 0.640637753 | 0.008584392 | 0.032459436 |
| FQU82_02303 | 564.8290084 | 0.68429727 | 0.008611971 | 0.032527775 |
| FQU82_03729 | 272.4212389 | 0.755955395 | 0.008980409 | 0.033733215 |
| FQU82_03762 | 210.7252166 | 0.651699335 | 0.009188972 | 0.034253446 |
| FQU82_02843 | 27.09279495 | 0.928340725 | 0.00922027 | 0.034332712 |
| FQU82_00961 | 59.39501913 | -0.589982336 | 0.009303015 | 0.034560437 |
| FQU82_01811 | 49.74797377 | -0.643190693 | 0.009331355 | 0.034560437 |
| FQU82_02602 | 105.5603843 | -0.757174863 | 0.009638603 | 0.035473172 |
| FQU82_03528 | 284.7067213 | 0.784652456 | 0.00975774 | 0.035750521 |
| FQU82_01363 | 29.233415 | -0.779040569 | 0.009815628 | 0.035890149 |
| FQU82_03582 | 44.88308534 | 1.146333036 | 0.010812375 | 0.038640317 |
| FQU82_03603 | 416.0676817 | 1.113021999 | 0.011004052 | 0.0390621 |
| FQU82_02641 | 295.5796744 | 0.587116238 | 0.011140533 | 0.039380255 |
| FQU82_02113 | 349.9953023 | 0.715755381 | 0.011185402 | 0.039419616 |
| FQU82_02030 | 13.91614284 | 1.244617223 | 0.011517234 | 0.040319612 |
| FQU82_01992 | 174.1506934 | -0.628942185 | 0.01160929 | 0.040414027 |
| FQU82_02077 | 30.77389452 | 0.762111583 | 0.012060217 | 0.041856047 |
| FQU82_00606 | 342.288554 | -0.65978253 | 0.012375057 | 0.042688955 |
| FQU82_02199 | 31.33935326 | 0.933375939 | 0.012699865 | 0.043633471 |
| FQU82_02219 | 100.8738435 | 0.594900509 | 0.012793988 | 0.043820924 |
| FQU82_01048 | 60.52886733 | -0.650054463 | 0.0132261 | 0.044945099 |
| FQU82_01682 | 31.68207713 | 0.703514919 | 0.013685756 | 0.046058356 |
| FQU82_01583 | 334.1487416 | 0.765443123 | 0.0137827 | 0.046239606 |
| FQU82_02141 | 49.21611334 | 0.591666964 | 0.013778917 | 0.046239606 |
| FQU82_00255 | 129.9251159 | 0.644523972 | 0.014431632 | 0.047853725 |
