# Supplementary material for: Transcriptomic analysis reveals the regulatory role of quorum sensing in the Acinetobacter baumannii ATCC 19606 via RNA-seq
Source: BMC Microbiol. 2022 Aug 16;22:198. doi: 10.1186/s12866-022-02612-z (PMC9380347; doi:10.1186/s12866-022-02612-z)
Supplement: Supplementary file 3 — Additional file 3: Figure S1. The PCA plot by ggplot2. The △abaI mutant and wild type (wt) were clustered into two groups based on the gene expression matrix. Figure S2. Heatmap of the Pearson correlation coefficient between the samples by corrplot. Figure S3. The map of the enriched KEGG pathways from the KEGG database[1, 2]. Green rectangles represented the downregulated genes and red rectangles represented the upregulated genes. Blue boxes represented the genes hyperlinked to KO entries, and white boxes represented the genes which were not hyperlinked to KO entries. (A) Phenylalanine metabolism (ko00360). (B) The type VI secretion system of bacterial secretion system (ko03070). Figure S4. The map of the enriched KEGG pathway “propanoate metabolism (ko00640)” from the KEGG database[1, 2]. Green rectangles represented the downregulated genes and red rectangles represented the upregulated genes. Blue boxes represented the genes hyperlinked to KO entries, and white boxes represented the genes which were not hyperlinked to KO entries. [file 12866_2022_2612_MOESM3_ESM.docx]

**Additional file 3**

**
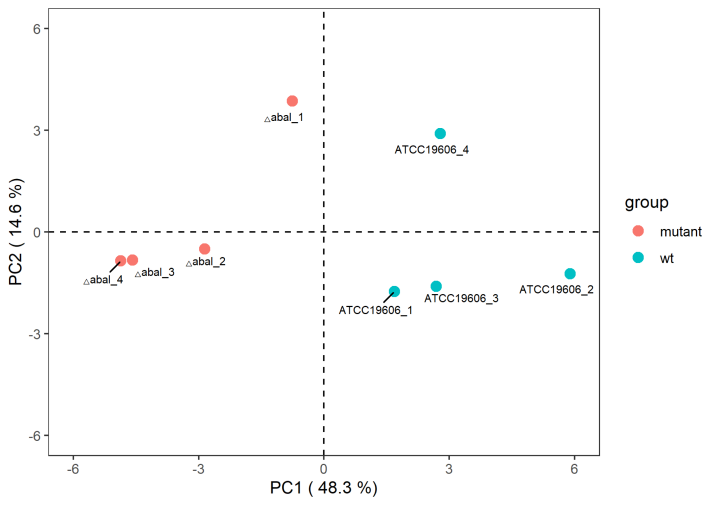
**

**Figure S1.** The PCA plot by ggplot2. The △abaI mutant and wild type (wt) were clustered into two groups based on the gene expression matrix.


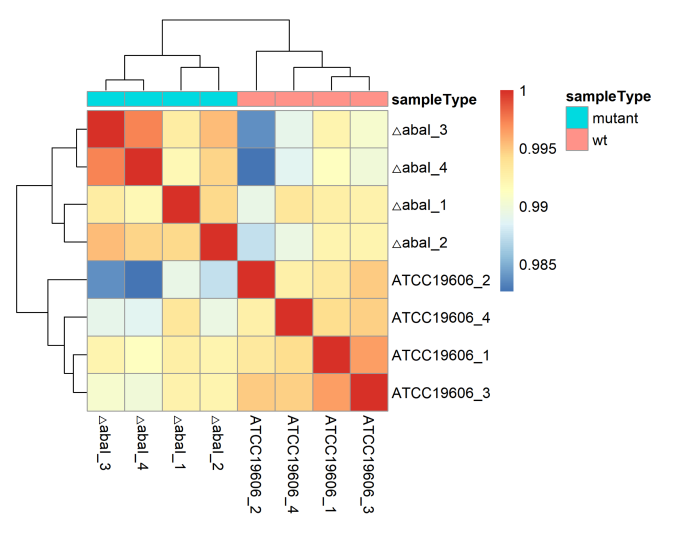


**Figure S2.** Heatmap of the Pearson correlation coefficient between the samples by corrplot.


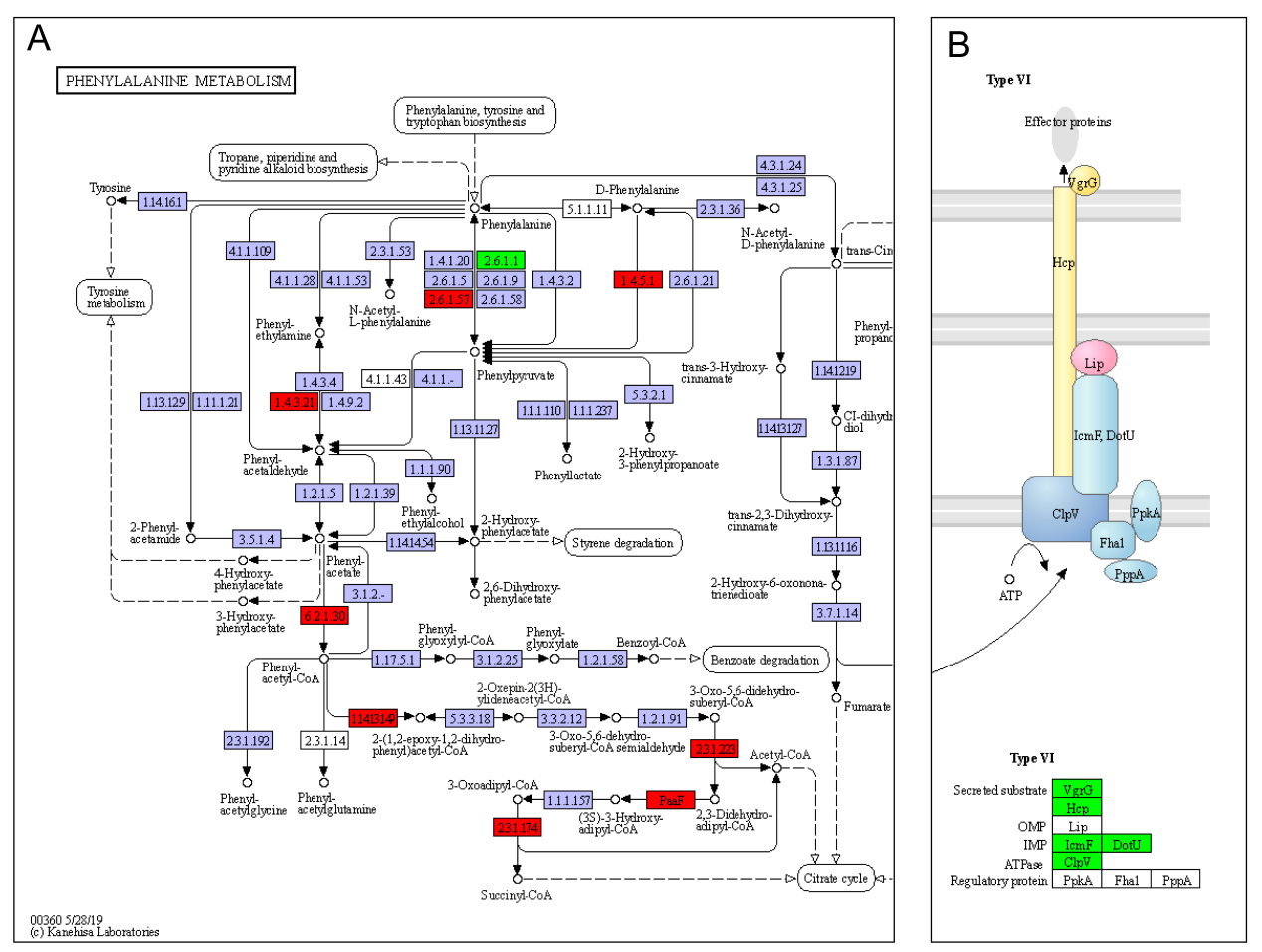


**Figure S3.** The map of the enriched KEGG pathways from the KEGG database[1, 2]. Green rectangles represented the downregulated genes and red rectangles represented the upregulated genes. Blue boxes represented the genes hyperlinked to KO entries, and white boxes represented the genes which were not hyperlinked to KO entries. (A) Phenylalanine metabolism (ko00360). (B) The type VI secretion system of bacterial secretion system (ko03070).


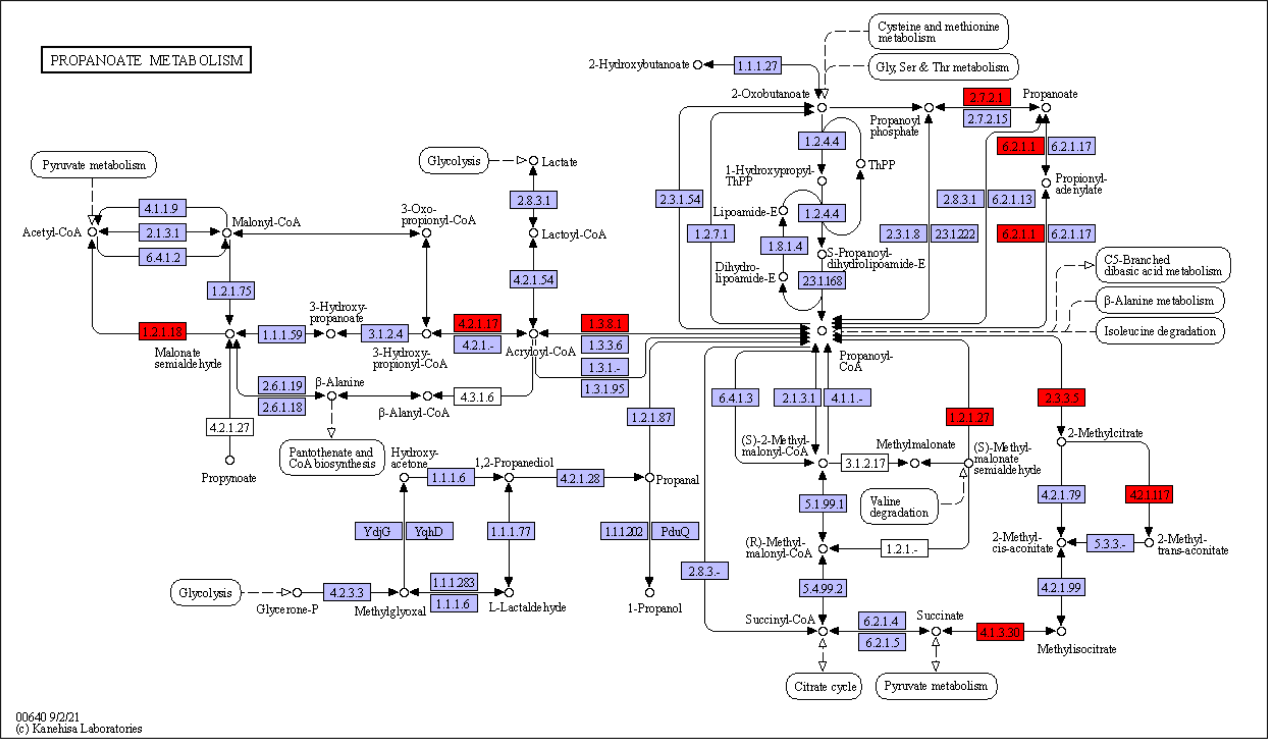


**Figure S4.** The map of the enriched KEGG pathway “propanoate metabolism (ko00640)” from the KEGG database[1, 2]. Green rectangles represented the downregulated genes and red rectangles represented the upregulated genes. Blue boxes represented the genes hyperlinked to KO entries, and white boxes represented the genes which were not hyperlinked to KO entries.

**References**

1. Kanehisa M, Goto S. KEGG: Kyoto Encyclopedia of Genes and Genomes. Nucleic Acids Res. 2000;28(1):27-30. <https://doi.org/10.1093/nar/28.1.27>.

2. Kanehisa M. Toward understanding the origin and evolution of cellular organisms. Protein Sci. 2019;28(11):1947-51. <https://doi.org/https://doi.org/10.1002/pro.3715>.
